# Effect of In Vitro Gastrointestinal Digestion on Amino Acids, Polyphenols and Antioxidant Capacity of Tamarillo Yoghurts

**DOI:** 10.3390/ijms23052526

**Published:** 2022-02-25

**Authors:** Tung Thanh Diep, Michelle Ji Yeon Yoo, Elaine Rush

**Affiliations:** 1 School of Science, Faculty of Health and Environment Sciences, Auckland University of Technology, Private Bag 92006, Auckland 1142, New Zealand; tung.diep@aut.ac.nz; 2 Riddet Institute, Centre of Research Excellence, Massey University, Private Bag 11222, Palmerston North 4442, New Zealand; elaine.rush@aut.ac.nz; 3 School of Sport and Recreation, Faculty of Health and Environment Sciences, Auckland University of Technology, Private Bag 92006, Auckland 1142, New Zealand

**Keywords:** tamarillo, yoghurt, fermentation, in vitro digestion, amino acids, polyphenols, antioxidant activity

## Abstract

Laird’s Large tamarillo powder is high in protein (10%) essential amino acids (EAAs), gamma-aminobutyric acid (GABA) and polyphenols (0.6% phenolics plus anthocyanins) and fibre 25%. This study aimed to investigate, using a standardized static in vitro digestion model, the stability of amino acids and antioxidant capacity of polyphenols in yoghurt fortified with 5, 10 and 15% tamarillo powder either before (PRE) or after (POS) fermentation. Compared to plain yoghurt, the fruit polyphenols (rutinosides and glycosides) were retained and substantial increases in FEAAs (free essential amino acids), total phenolic content (TPC) and antioxidant activity were observed particularly at the end of intestinal phase of digestion. Together with SDS-PAGE results, peptides and proteins in tamarillo yoghurts were more easily digested and therefore may be better absorbed in the small intestine compared to the control. TPC and antioxidant activity of fortified yoghurts increased significantly after in vitro digestion. Relatively high bioaccessibilty of chlorogenic acid and kaempferol-3-rutinoside in digested PRE samples was observed. The results suggest that the yoghurt matrix might protect some compounds from degradation, increasing bioaccessibility and in the small intestine allow increased absorption and utilization possible. Fortification would deliver intact polyphenols and fibre to the large intestine and improve gut health. Further research of acceptability, shelf life, and then trials for health effects should be implemented.

## 1. Introduction

The development of new food products with a potentially positive effect on health using traditional fruits, is generally desirable since there is an increasing interest among consumers to look for safe, healthy, sustainable and natural foods [1]. Consumption of fruits is associated with health benefits which are usually related to their vitamin, mineral or specific antioxidant compounds, in particular, polyphenols which possess strong antioxidant activity and are associated with protective effects against chronic diseases including type 2 diabetes mellitus, cardiovascular diseases and cancer [2].

We have previously shown that the protein content of New Zealand-grown Laird’s Large cultivar of tamarillo is 1.2% fresh weight (FW) (88.1% moisture content) which is equivalent to 10% dry weight (DW) [3] (Supplementary Table S1). In addition, tamarillo powder has a high fibre content (25%), essential for bowel health. Twenty-two amino acids (9 essential and 13 non-essential were detected [3]. Tamarillo showed a high concentration of γ-aminobutyric acid (GABA) (433 mg/100 g DW) which is similar to tomato, the same *Solanum* genus [3]. In addition, tamarillo fruit contains a spectrum of polyphenol components including not only the blue-red coloured anthocyanins; delphinidin-3-rutinoside (254.76 mg/100 g DW) and pelargonidin-3-rutinoside (200.66 mg/100 g DW), but also the colourless phenolics, particularly chlorogenic acid (66.35 mg/100 g DW) and kaempferol-3-rutinoside (50.04 mg/100 g DW) contributing to high antioxidant activity (52.42 and 60.19 μmol TEAC/g DW determined by CUPRAC and FRAP assays, respectively) [4]. It has been reported that tamarillo polyphenols have shown promising effects including antioxidation and antioxidative stress [5], anti-obesity [6], anticancer [7,8], anti-microbial activity [9] as well as protection against lipid oxidation [10].

Among the principal issues concerning the beneficial effects of amino acids and polyphenols, their bioavailability and metabolic pathway must be considered. The bioavailability of a dietary compound is dependent on its release from the food matrix (referred to as bioaccessibility) based on particle size and form [11], its digestive stability, and the efficiency of its transepithelial passage. In particular, bioavailability differs greatly from one polyphenol to another, and can depend on the dietary source [12]. Research concerning the bio-accessibility of polyphenols from food matrices is important, since only the compounds released from the food matrix are potentially bio-accessible, and after the gastrointestinal digestion, are in a condition to exert their beneficial effects [12].

In the food matrix, proteins are known to reversibly and irreversibly conjugate with polyphenols in different ways dependent on characteristics of the proteins, the polyphenols, the food matrix and the stage of digestion [13]. These characteristics include solubility, pH, thermal stability, non-covalent bonding and enzymatic reactions [13]. Protein-polyphenol complexes may improve the stability of food emulsions on the shelf but also be a mechanism to prevent degradation of the polyphenol and target delivery to the intestinal tract.

Yoghurt is the most popular fermented dairy product [14] and is highly appreciated for its nutritional value and good digestibility. The health benefits of yoghurt have been recognized due to the presence of bioactive peptides and probiotics [15]. However, plain yoghurt is not considered a source of polyphenols and therefore traditional foods such as spices, fruits, seed or extract had been used to enhance the polyphenol content of yoghurt. Many studies have attempted to investigate the effect of plant extracts in order to improve the quality of yoghurt such as increasing antioxidant activity and the viability of lactic acid bacteria during refrigerated storage [16]. The yoghurt matrix is an excellent delivery vehicle for plant-derived polyphenol compounds. The low pH of yoghurt increases the stability of phenolic compounds during storage [15], whereas the presence of proteins or large peptides and fats maintain the integrity of polyphenols during digestion, increasing their bioaccessibility [17].

The main aim of the current study was to fortify yoghurt using tamarillo powder and to evaluate how amino acid, polyphenol concentrations and the antioxidant activity are affected during digestion. For this purpose, six tamarillo yoghurts (produced from pre- and post-fermentation processes at three different levels of fortification) and a control sample were examined for the successive effects of in vitro digestion at oral, gastric and intestinal phases on amino acid, polyphenol concentrations and antioxidant activity.

## 2. Results and Discussion

### 2.1. Protein Fractions by Mass

**Figure 1:**
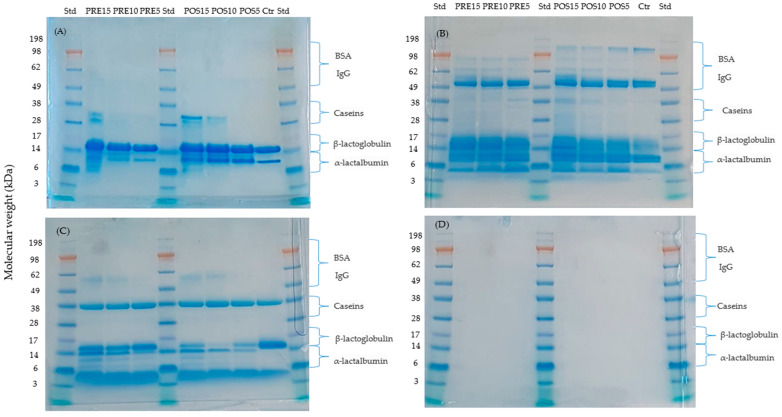
Representative SDS-PAGE images of proteins profile of control and tamarillo yoghurts during the gastrointestinal digestion simulation. (**A**): undigested sample, (**B**): oral phase, (**C**): gastric phase, (**D**): intestinal phase. Numbers on *y* axis represent molecular weight in kDaltons (kDa). Lanes from left to right: standard, PRE15, PRE10, PRE5, standard, POS15, POS10, POS5, control and standard, respectively. POS5, POS10 and POS15: 5%, 10%, 15% of tamarillo powder was added post fermentation, respectively. PRE5, PRE10 and PRE15: 5%, 10%, 15% of tamarillo powder was added to milk and starter culture prior to fermentation, respectively. Ctr: Control, Std: Standard.

At a molecular level, marked differences in the protein fractions in control and tamarillo yoghurts before and after oral, gastric and intestinal phases of digestion (Figure 1) were observed. Prior to digestion, all yoghurts including the control (Figure 1A) had lower molecular weight (MW) whey proteins (α-lactalbumin and β-lactoglobulin) present. The caseins band was only observed in yoghurts fortified with 10% and 15% tamarillo powder. The intensity of β-lactoglobulin, the predominant component of whey protein, was similar for all yoghurt samples indicating that this compound was not degraded with or without addition of tamarillo powder. Meanwhile, α-lactalbumin showed higher intensity for control and the tamarillo yoghurts produced from the post-fermentation process rather than yoghurts fortified in pre-fermentation. These changes most probably were due to proteolysis induced by tamarillo protease during fermentation [18]. Hence, fermentation degraded α-lactalbumin but not the major whey proteins. This is similar to the changes in the protein profile observed 24 h after a strawberry preparation was added to fermented yoghurt [19].

The proteins fractions in the yoghurt after the oral phase of digestion (Figure 1B) differed from the undigested yoghurts. Bands in the low molecular mass region (α-lactalbumin and β-lactoglobulin) were observed after the oral phase, corresponding to peptides produced by hydrolysis. A higher intensity of caseins was detected in tamarillo yoghurts with higher concentration of fortification (10% and 15%) compared to the 5% tamarillo yoghurts or control sample. In the high molecular mass region (>98 kDa), the control and tamarillo yoghurts produced from post-fermentation showed higher intensity bands than the pre-fermentation fortified yoghurts, confirming for more extensive proteolysis in PRE samples as shown in undigested samples.

Gastric digestion (Figure 1C) resulted in further reduction in higher molecular weight proteins (>38 kDa) and an increase in lower molecular weight protein and/or peptides than the β-lactoglobulin. Whey protein was hydrolyzed by simulated gastric acid and pepsin during the 120 min of gastric digestion. Meanwhile, β-lactoglobulin was not significantly different (*p* > 0.05) among the yoghurts produced from either pre- or post-fermentation process. The band of β-lactoglobulin was thicker for the control yoghurt than fortified samples, and some undefined bands appeared for tamarillo yoghurts but not for the control sample. For all yoghurt samples, only one casein band which was very thick, appeared after the gastric phase in contrast to several thin bands appearing in the oral phase. It was proposed that this band could represent gastric curd associated with α-casein with a molecular weight of 38 kDa [20]. Others have shown that pasteurization of the milk used in yoghurt slows the rate of hydrolysis of protein in the gastric phase [21] but we could not find any literature reporting the effect of thickening of yoghurt with fruit powder on the nature of the curd and protein digestion in the stomach. Faint bands still appeared in high molecular weight region (BSA and IgG with MW of 66.5 and 150 kDa, respectively) for yoghurt fortified with high concentration of tamarillo powder (10% and 15%). This suggests different kinetics of protein release among yoghurt matrices thickened with higher concentrations of tamarillo powder and low concentration or without tamarillo powder. We have previously shown that the addition of tamarillo to yoghurt reduces syneresis and increases stiffness of the yoghurt matrix [22].

Following the 180 min of intestinal digestion, the patterns were similar for all yoghurt samples (Figure 1D). No bands were observed at the bottom of the gel, suggesting that proteins and large peptides (>7 kDa) were further hydrolyzed into small peptides (<7 kDa) and amino acids and therefore not detected. Lorieau et al. [23] stated that the proteolysis to amino acids and peptides only begins with the addition of the pancreatin enzymes at the beginning of the intestinal phase. Additionally, this observation could be related to the appearance of several bands of smaller proteins below the α-lactalbumin band and peptides around 3 kDa. At the end of the intestinal phase, soluble caseins were totally degraded in all yoghurt samples, which was in line other studies [21,24]. 

The current results are in agreement with Sousa et al. [25]. These authors observed that in whey protein isolate, serum albumin (64 kDa) hydrolysis occurred at the beginning of the gastric phase and was no longer present at the end of this phase. Meanwhile, β-lactoglobulin was resistant to pepsin in the gastric phase and immediately hydrolyzed at the beginning of the intestinal phase. The rate of protein digestion has been attributed to three factors: (i) hydrolysis by hydrochloric acid and pepsin; (ii) dilution of the digesta by the incoming gastric fluid during digestion; (iii) the size and denaturation of protein particles [26]. The reduction in concentration of β-lactoglobulin was probably due to dilution by the gastric fluid as the reduction in β-lactoglobulin was close to or slower than the dilution of protein. However, the reduction in α-lactalbumin may have resulted from both dilution and pepsin hydrolysis, especially at long digestion times (beyond 160 min), when the pH was lower than pH 4.

### 2.2. Effect of In Vitro Digestion on Free Amino Acid Profiles of Tamarillo Yoghurts

Milk protein is a complete protein containing all essential amino acids that are required for human growth and development. Consumption of extra plant proteins could further help to increase the intake of essential amino acids [27], which was observed from this study with higher essential free amino acid (EFAA) in tamarillo fortified yoghurt than the control (Supplementary Table S2A and Table 1). Tamarillo powder which contains 80% or more protein by weight, make it possible to consume 10–20 g or more of plant-based protein in one serving of a ready-to-drink shake or powder mix.

The EFAA profile of PRE and POS yoghurts were dominated by isoleucine and histidine, respectively. The addition of tamarillo powder to the yoghurts pre and post fermentation (but before any digestion) was associated with substantial and dose-related increases in total free essential (up to 72×) and non-essential amino acids (up to 106×) (Table 1) in the yoghurt suggesting proteolytic activity of the tamarillo powder during and after fermentation (Supplementary Table S2A shows individual amino acids). The total EFAAs of PRE was higher than for POS fermentation techniques. This might be due to the breakdown of protein during the fermentation process that led to increase in EFAA content in PRE samples.

After the intestinal digestion phase, the effect of the addition of tamarillo powder and the increase in total free amino acids (TFAAs) was clear (Table 1). Before digestion, the lowest concentrations of TFAAs were seen in the control and the highest in the 15% tamarillo yoghurt. After intestinal digestion, the TFAAs of control increased 200× and the TFAAs in the 15% POS yoghurt increased 4×. The difference between the control and POS15 samples was possibly due to proteolysis induced by tamarillo protease which has been observed in a previous study [18]. Our previous study [22] has also shown the benefits of fortified yoghurt with tamarillo powder including not only maintaining the distinctive flavor-associated volatiles from the fruit but also increasing the total acidity, fibre, protein and lactic acid contents of yoghurts. We have shown that yoghurt fortified with tamarillo powder could be a dietary source of α-tocopherol and β-carotene as well as a good dietary source of vitamin C [22].

There is concern that some fruit yoghurts may exhibit shorter shelf life and unfavorable flavor characteristics arising from the proteolysis with the addition of fruit. On the other hand it is important to enhance the digestibility of protein and take the FAAs present in the ileum into consideration [28].

In the tamarillo yoghurts compared with control, the increased availability after intestinal digestion of EFAAs including L-glutamic acid, γ-aminobutyric acid (GABA) and L-aspartic acid, is of importance (Table 2). In response to the low bioavailability of GABA (produced from L-glutamic acid) in dairy products, researchers have used proteolytic co-culture of yoghurts using starters and the enzyme trypsin [29].

The concentration of each FAA increased from oral to intestinal in vitro digestion, that led to a gradual increase in total free amino acids (TFAAs), total essential free amino acids (TEFAAs) and total non-essential free amino acids (TNEFAAs) (Supplementary Table S2B and Table 1) for the same route. After intestinal digestion the TFAAs and TNEFAAs in fortified yoghurts with 15% tamarillo power added were approximately double and 2.4–3 times than control sample, respectively (Table 1). The profiles of EFAA and TFAA of tamarillo yoghurts after intestinal digestion produced from pre and post fermentation process were quite similar (Table S2B). In the control yoghurt, the TFAAs level increased to about 11 and 3 times by the end of intestinal phase compared to the oral and gastric phases, respectively. The TFAAs level increased to approximately 2.5–4.6 and 2 times by the end of intestinal phase compared to the oral and gastric phases, respectively, for tamarillo yoghurts (Supplementary Table S2B and Table 1). This result is in agreement with the SDS-PAGE result where no protein fragments were detected in intestinal phase indicating potential absorption of amino acids within 5 h. Therefore, it can be assumed that peptides and proteins in tamarillo yoghurts have been completely digested and free amino acids could be absorbed in this time gastrointestinal tract. It is in coherent with the high digestibility of milk proteins, which are the most degradable proteins among food proteins with an ileal digestibility of 95% and 97% for caseins and whey proteins, respectively [30].

The current finding indicated that the main role of gastric pepsin was to break down large proteins into smaller fragments which would be ready for the more complete digestion by pancreatic enzymes and the concomitant release of FAAs. Pancreatic enzymes contribute about 2–4 times more than pepsin to proteolysis during human gastrointestinal digestion [31] similar to the magnitude observed in the current study (Supplementary Table S2B). Several FAAs (L-alanine, L-glycine, L-isoleucine, L-leucine, and L-valine) could be produced as a result of further digestion by pancreatic endo- and exopeptidases [31] which may explain the significant increase in these FAAs in yoghurts after pancreatic digestion.

For the oral phase, concentration of FAAs was significantly different among different yoghurt samples (*p* < 0.05). The control yoghurt showed the highest release rate of TFAAs, TEFAAs and TNEFAAs with 16, 51 and 6.6 times compared to undigested samples. The TEFAAs increased 3.1–7.4 and 1.3–2.7 times for fortified yoghurt produced from post- and pre-fermentation process, respectively. The TFAAs and TNEFAAs of both POS and PRE samples just increased 1.2–2.3 times compared to undigested samples. At the oral phase, L-glutamic acid was the highest FAA in all fortified yoghurts whereas, L-Lysine was the highest FAA in control sample after oral phase. There was a dose-dependent relationship between the amount of EFAAs and concentration of tamarillo powder added. Higher amount of most FAAs as well as TFAAs, TEFAAs and TNEFAAs (approximately 1.4–2.8 times) were observed in yoghurts after the gastric phase compared to the oral phase. This was due to the shorter reaction time of oral phase (5 min) while the gastric phase took 2 h. After the intestinal phase, the key amino acids, L-glutamic acid, L-aspartic acid and GABA, in all fortified yoghurts was higher than control (Table 2). L-isoleucine was observed as the highest level in control sample (Supplementary Table S2B). Since GABA is a non-protein amino acid, i.e., not a substrate for the digestive enzymes used in the study, relatively similar concentrations in fortified yoghurts after digestion were observed (Table 2).

Bioavailability and concentrations of amino acids in the intestine has a potential effect on protein metabolism at the splanchnic and peripheral levels. For example, leucine is known to stimulate the muscle protein synthesis [30] and the concentration of this AA increased 6–8.5 and 28–95 times after intestinal digestion compared to undigested samples for PRE and POS, respectively (Supplementary Table S2A,B). Therefore, ingestion of tamarillo yoghurts may help to induce muscle protein synthesis. The present study thus contributes to the importance of food matrix design for the control of nutrients delivery.

Quantification of FAAs has been affected by chemical reactions, oxidation or hydrolysis. For example, due to oxidation, methionine sulfone and cysteic acid have been quantified rather than methionine and cysteine, respectively. Accurate measurement of oxidation rate was difficult. Partial degradation (5–10%) during hydrolysis have been reported for serine and threonine [28]. As observed by [28], a decrease in uptake of phenylalanine and tryptophan was caused by increase in branched-chain amino acids, due to competition for the same transporter. As amino acids undergo structural changes during digestion, their availability for protein synthesis may also be affected. For example, methionine could be oxidized, and the oxidized derivatives may be either poorly utilized or not nutritionally available. The determination of both the amount and the profile of EFAAs absorbed from a protein was related to determination of the individual values of digestibility in the current study, following Wolfe et al. [28].

### 2.3. Effect of In Vitro Digestion on Total Phenolic Content and Antioxidant Activity of Tamarillo Yoghurts

**Figure 2:**
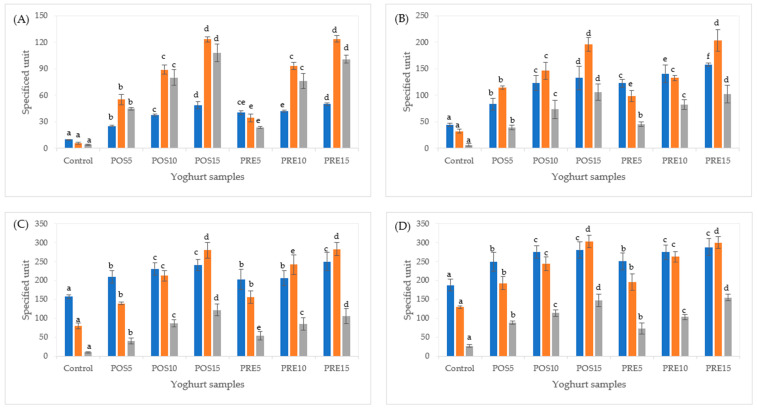
Total phenolic content (TPC) and antioxidant activity (CUPRAC and FRAP assays) of yoghurts fortified with tamarillo powder before digestion (**A**), after oral (**B**), gastric (**C**) and intestinal (**D**) phases of in vitro digestion. TPC (■) (mg/100 g yoghurt), CUPRAC (■) (mg TEAC/100 g yoghurt) and FRAP (■) (mg TEAC/100 g yoghurt). Data are presented as mean and error bar (standard deviation) (n = 3). Different alphabets indicate statistical difference (*p* < 0.05) for each assay. POS5, POS10, and POS15: 5%, 10%, and 15% tamarillo powder was added post-fermentation, respectively. PRE5, PRE10, and PRE15: 5%, 10%, and 15% tamarillo powder was added to milk and starter culture prior to fermentation, respectively.

Fortified yoghurts using pre- or post-fermentation approach had higher TPC values (2.5–5 times) and antioxidant activity (82–95%) than the control yoghurt (*p* < 0.05) (Figure 2A), suggesting that the added polyphenols from tamarillo had been retained. Higher level of tamarillo fortification led to a higher TPC as well as antioxidant activity of yoghurts. The undigested PRE samples showed higher TPC value than POS with 37%, 10% and 2% with addition of 5%, 10% and 15% tamarillo added, respectively. The polyphenols from tamarillo added in pre-fermentation might be released from protein-phenol conjugates and become more extractable [32]. This might be because of the catabolic action of enzymes in the tamarillo [18] and also produced during fermentation [33]. Additionally, the activities of β-glucosidase would be able to hydrolyze conjugated phenolics to release free phenolics [34]. According to Adebo and Gabriela Medina-Meza [35], increased extractability of polyphenols might be due to structural breakdown of cell walls after fermentation. The breakdown of the cell wall also led to the release of various bioactive compounds which cause increase TPC. The TPC of control was most likely related to the presence of milk compounds that are not polyphenols such as low molecular weight antioxidants (<900 Da) such as organic acids, fatty acids, vitamins and minerals as well as free amino acids, peptides and proteins that interfere with the Folin–Ciocalteu reagent [15]. Similar results had been observed for yoghurt fortified cinnamon powder [15] and *Rhus coriaria* leaf powder [36]. The antioxidant activity of control is mainly due to the formation of bioactive peptides with radical scavenging activity because of the proteolytic activity of *Lactobacilli* in the starter culture. The POS5 showed higher CUPRAC and FRAP values than the PRE5, while with 10% and 15% tamarillo added, relatively similar antioxidant activity between POS and PRE samples were observed.

At neutral pH (oral and intestinal phases), the PRE samples showed higher TPC than POS ones (Figure 2B,D); whereas at acidic pH (gastric phase), similar TPC value between PRE and POS was observed for 5% and 15% tamarillo added (Figure 2C). According to Simonetti et al. [36], at yoghurt pH (4.5), the binding affinity between polyphenols and milk proteins is enhanced which may lead to a decrease in the digestibility and absorption of nutrients and bioactives from yoghurt. However, during in vitro digestion, hydrolytic enzymes and pH changes lead to hydrolysis of proteins and/or peptides, resulting in the release of polyphenols that were bound, and therefore this would increase their bioaccessibility. Pepsin at acidic pH (gastric phase) would digest majority of the proteins to polypeptides thus interrupting the protein–polyphenol interaction and resulting in the release of more free polyphenols into the digestive fluid; whereas, pancreatin at neutral pH (intestinal phase) would complete the hydrolysis to smaller peptides and some amino acids [36]. In line with this, the TPC after in vitro gastric digestion represented about 84% and 75–87% of the TPC released after complete in vitro digestion for control and fortified yoghurts, respectively. A similar trend was observed in yoghurt fortified with strawberry and peach [12], cinnamon [15] and *Rhus coriaria* leaf powder [36]. After pancreatic digestion, the TPC of fortified yoghurts shows an increase in phenolic content compared to the undigested yoghurt of about 6–10 times. According to Simonetti et al. [36], Folin–Ciocalteu method detects the free polyphenols from tamarillo, the endogenous milk phenols derived from animal feed, and nonphenolic compounds from milk such as free amino acids and peptides that interfere with the Folin–Ciocalteu reagent. However, this method is not able to detect the polyphenols fraction that remains linked to milk components such as protein, lipids, and carbohydrates. Additionally, the accuracy of this assay can be affected by several compounds including ascorbic acid, dehydroascorbic acid (DHA), and reducing sugars (glucose and fructose) [37]. Ascorbic acid and dehydroascorbic acid (both enediols) rapidly react with polyphosphotungstate under acidic pH of the reagent, showing a blue color right after mixing the extract with the reagent. Under the alkali conditions of the assay, enediol reductones from reducing sugars are formed and react with the reagent. Therefore, the results for TPC determinations are skewed [37]. To get a better antioxidant activity profile of yoghurts, three assays (Folin, CUPRAC and FRAP) were used in the current study. This was because FRAP, CUPRAC, and Folin methods can be used for acidic (pH 3.6), neutral (pH 7.0), and alkaline (pH 10) media, respectively [38].

The difference between TPC identified by the Folin–Ciocalteu method mgGAE% (Figure 2) and concentration of polyphenols determined by LC-MS/MS mg% (Table 3 and Table 4) had been observed in our previous study [4]. The Folin–Ciocalteu colorimetric method is a measure of the reduction capacity of phenolic compounds, but free amino acid, fatty acids, organic acid, and vitamins (A, C and E) may interfere. Whereas the LC-MS/MS directly quantified specific polyphenol compounds including glycosylated or ester-linked groups. Additionally, possible explanations for the ambiguous relationship between TPC and polyphenol concentration is that synergy in a mixture makes TPC not only dependent on polyphenol concentration but also on the structure and interactions among polyphenols [35]. Hence, directly linking TPC in food and responsible components might be somewhat difficult, as methods of extraction, identification, and/or quantification of TPC and individual polyphenol vary, which lead to difficulty in drawing comparisons and, subsequently, extrapolating conclusions [35].

The antioxidant activity of yoghurts (CUPRAC and FRAP values) was associated with polyphenols content and as expected, after each phase of in vitro digestion, it increased significantly (*p* < 0.05) (Figure 2B–D). Yoghurts fortified with strawberry and peach [12], cinnamon [15] and *Rhus coriaria* leaf powder [36] have shown a similar trend. Antioxidant activity in polyphenol fortified dairy products increased during digestion as a result of peptic and pancreatic enzyme activity, breaking down the protein structures. These enzymes promoted the release of both polyphenols bound in protein–polyphenol complex and bioactive peptides and amino acids bound in milk protein sequences [39]. After the gastric phase, CUPRAC and FRAP values of fortified yoghurts corresponded to an increase of over 72% and 67% compared to the same values after intestinal phase, respectively, except for FRAP value of POS5 with 45%. It can be concluded that the acidic pH together with enzymes action led to both higher polyphenol extractability from protein–polyphenol complexes and release of bioactive peptides. After the intestinal phase, the CUPRAC and FRAP values of fortified yoghurts increased about 2.4–3.5 and 1.4–3.1 times, respectively, compared to the undigested samples. The same antioxidant activity trend was observed by others in yoghurts fortified with strawberry and peach (ORAC assay) [12] as well as *Rhus coriaria* leaf powder (ABTS and FRAP assays) [36].

### 2.4. Effect of In Vitro Digestion on the Polyphenol Profile of Tamarillo Yoghurts

In the fortified yoghurts prepared using the pre- or post-fermentation approach, all polyphenol compounds from tamarillo fruit [4] were detected (Table 3), thus, the yoghurt matrix has helped retain these individual polyphenols during fermentation. Compared to the estimated contributions of tamarillo powder to nutrient and phytochemical content of fortified yoghurt (Supplementary Table S1), the actual concentration of main polyphenols (Table 3) as well as TPC, CUPRAC and FRAP values (Figure 2A) in all undigested tamarillo yoghurt samples were lower or relatively similar except for chlorogenic acid (in POS5) and delphinidin-3-rutinoside (in POS5 and PRE5) with higher concentrations were overserved. The increase in tamarillo powder added led to higher amount of polyphenols in fortified yoghurts (*p* < 0.05). As expected, chlorogenic acid, kaempferol-3-rutinoside, delphinidin-3-rutinoside and pelargonidin-3-rutinoside (which account for over 90% of the total polyphenol content in yoghurts) showed high levels of all the identified polyphenol compounds in the fortified yoghurts. Chlorogenic acid showed relatively similar concentrations between POS and PRE samples for all fortified yoghurts. For kaempferol-3-rutinoside and pelargonidin-3-rutinoside, POS5 and POS15 showed higher concentration than the PRE5 and PRE15, whereas POS10 and PRE10 have had the same concentration of these polyphenols. Similar level of delphinidin-3-rutinoside was observed for POS and PRE samples with 5% and 10% tamarillo added, while PRE15 showed a lower content of this compound than the POS15. This could be explained by the yields of polyphenols identified by LC-MS, which were related to the extractability of polyphenols from the food matrix and the stability of these polyphenols during food processing [32]. Polyphenol extractability might also be influenced by chemical and physical effects such as the gel structure of yoghurt, binding to amphipathic yoghurt peptides or complexation with proteins and polysaccharides [32]. The high level of chlorogenic acid in both POS and PRE samples suggested that chlorogenic acid was not metabolized by the starter cultures or was not degraded into other molecules, regardless of addition approach. Similar trend had been observed in yoghurt fortified with apple polyphenols by Sun-Waterhouse, Zhou and Wadhwa [40]. These authors concluded that the fermentation process showed less impact on chlorogenic acid than other polyphenols. Acidity of yoghurt induced acid hydrolysis of polyphenols, hence low level of flavonol compound (kaempferol) in yoghurts has been reported for fortified yoghurts [40].

As shown in Table 3 and Table 4, different behaviours of identified polyphenols before and after in vitro digestion were observed. Major polyphenols (chlorogenic acid, kaempferol-3-rutinoside, delphinidin-3-rutinoside and pelargonidin-3-rutinoside) in fortified yoghurts showed high concentration at the end of each digestion phase compared to other polyphenol compounds (*p* < 0.05). After in vitro digestion, chlorogenic acid represented about 44–54% and 42–71% of this compound concentration in undigested POS and PRE samples, respectively, indicating relatively high bioaccessibilty of this polyphenol in tamarillo yoghurt. A chlorogenic acid level of 61% after in vitro digestion compared to undigested sample had been observed in peach yoghurt [12], owing to chlorogenic acid forming stable milk casein complexes under simulated gastrointestinal conditions [36]. During in vitro digestion, the oxidation and polymerization could cause the degradation of chlorogenic acid to form quinone [17]. For PRE samples, the percentage ratio between kaempferol-3-rutinoside content in post-pancreatic digested and undigested samples was over 65%, while the number for POS sample was 31–50%. This might be due to kaempferol-3-rutinoside being broken down into smaller forms that would be more extractable, when added prior to fermentation.

A similar phenomenon was observed for caffeic acid with over 75% and 20–43% for PRE and POS samples, respectively. For all fortified yoghurts, the percentage ratio of catechin and epicatechin in digested and undigested samples was below 20% and above 75%, respectively. This may be explained by epicatechin having lower interactions with α- and β-casein than catechin, in terms of lower binding constant value and number of polyphenols bound for casein molecule. This would result in a less protection of epicatechin and therefore, a greater susceptibility to the enzymes action, which can be detected with LC-MS analysis [41]. Additionally, lower release of catechin during digestion is due to a ring structure in the molecule that facilitates dissolution in milk fat globule membrane [42]. After the pancreatic digestion, rutin showed a percentage ratio of 51–63% and 45–83% between digested and undigested for POS and PRE samples, respectively (Table 3 and Table 4). The percentage ratio of rutin in yoghurt fortified with strawberry, peach and *Rhus coriaria* leaf powder was 60%, 67% and 52%, respectively [12,36]. According to Simonetti et al. [36], rutin, with low dissolution rate and bioavailability, forms complexes only with the bovine serum albumin and the β-lactoglobulin, while the free rutin can undergo several chemical and enzymatic degradation steps in the gastrointestinal environment.

The percentage ratio for delphinidin-3-rutinoside content between post-pancreatic digested and undigested samples was 23–38% and 6–14% for PRE and POS samples, respectively (Table 3 and Table 4). For pelargonidin-3-rutinoside, the ratio was 16–34% and 18–21% for PRE and POS samples, respectively. The transition from the acidic gastric to the mild alkaline intestinal environment caused a decrease in the amount of bioaccessible anthocyanins [12]. High instability of anthocyanins at neutral or slightly basic pH due to the formation of the colourless chalcone pseudo-base has resulted in the destruction of an anthocyanin chromophore, as reported by [43,44,45,46]. Anthocyanins from strawberry have been reported as highly stable under the acidic conditions of the stomach while they have been degraded under the alkaline conditions of the intestine [12]. The same finding has been observed in this study (Supplementary Table S3A,B).

Strengths of the current study are that this is the first study to fortify yoghurt with tamarillo powder, either pre-fermentation or post-fermentation and effect of in vitro digestion on amino acids, polyphenols and antioxidant activity was examined. This study highlights after simulated gastro-pancreatic digestion the high concentrations and bioaccessibility of diverse free amino acids, free polyphenols, and antioxidant activity, in yoghurt fortified with tamarillo powder. Our results have demonstrated that tamarillo pulp powder can be used to fortify yoghurt with essential amino acids, GABA and polyphenols. The results suggest that the yoghurt matrix allowed the protection of some compounds from degradation increasing bioaccessibility and making absorption and utilization possible. In fortified yoghurts, polyphenol compounds released from the yoghurt were stable in the digestive environment, thus would be able to exert their biological effects on the gastrointestinal system, which is more important than the content of these compounds in the corresponding undigested food. For example, chlorogenic acid in milk casein complex form has shown stability under simulated gastrointestinal conditions [47]. Additionally, high stability of chlorogenic acid and caffeic acid under digestive conditions has been reported [48]. However, to obtain a better understanding on polyphenols bioaccessibility from tamarillo-fortified yoghurt, further in vivo studies evaluating both the action of digestive enzymes and the action of microbiota metabolism should be performed. Additionally, the changes of polyphenols in freeze-dried tamarillo pulp only (as a control) during in vitro digestion should be implemented to highlight the possible protective effects operated by yoghurt on polyphenols.

## 3. Materials and Methods

### 3.1. Materials

Yoghurt ingredients were standard milk (Anchor™ blue top, 3.3% protein and 3.4% fat) from Fonterra, New Zealand and starter culture, *Lactobacillus delbrueckii subsp. bulgaricus* and *Streptococcus thermophilus* (YoFlex^®^ Express 1.1 powder) from CHR Hansen (Hoersholm, Denmark). Freeze dried tamarillo (Laird’s Large cultivar) powder was used for fortification.

All chemicals and reagents used were AnalaR grade or purer. The analytical grade standards of amino acids standards (A9906 product), phenolics and anthocyanins were obtained from Sigma-Aldrich (Auckland, New Zealand) or Extrasynthese (Genay Cedex, France). All chemicals and gels for SDS-PAGE experiment including LDS Sample Buffer (4×), MES Running Buffer, Coomassive Blue R-250, Bis-Tris Protein Gels and Prestained standard were obtained from Thermo Fisher (Auckland, New Zealand). All chemicals for total phenolic content (TPC) and antioxidant activity were purchased from Sigma-Aldrich (Auckland, New Zealand). Milli-Q water was produced by a Purite Fusion Milli-Q water purifying machine (Purite Limited, Thame, Oxon, UK).

### 3.2. Yoghurt Preparation

Yoghurt samples were prepared using commercial yoghurt makers (Davis & Waddell, Steven, New Zealand). For the control yoghurt, starter culture and milk in the ratio of 0.1:100 (*w*/*w*), were placed in the yoghurt maker and set at 45 °C for 8 h and until the pH dropped below 5.0. The yoghurt was stored at 4 °C overnight and then homogenized at 4000 RPM (L4R Laboratory Mixer, Silverson, Waterside, England) for 2 min [49].

Freeze-dried tamarillo pulp powder (5%, 10% and 15%) was added into the yoghurt either before (PRE) or after (POS) the fermentation process. For PRE, prior to fermentation, tamarillo powder was added to the mixture of milk and starter culture at the start of the yoghurt making process. For POS, tamarillo powder was added to the yoghurt after fermentation in the final homogenization step. Counting the control yoghurt without tamarillo, seven different formulations in total were prepared for digestion. Calculated total protein content of each mixture was 3.3% for control [50], 3.6% for 5%, 4.0% for 10% and 4.3% for 15% as each 5% of tamarillo powder contributed 0.5% of tamarillo protein [3]. The polyphenol compounds in tamarillo powder have been identified in our previous study [4], and these were *p*-coumaric acid, caffeic acid, catechin, epicatechin, chlorogenic acid, ellagic acid, ferulic acid, gallic acid, kaempferol, rutin, kaempferol-3-rutinoside, isorhamnetin-3-rutinoside, cyanidin-3-rutinoside, delphinidin-3-rutinoside and pelargonidin-3-rutinoside with the concentration of main polyphenols has been described in Supplementary Table S1.

### 3.3. In Vitro Digestion of Yoghurt

The static in vitro enzymatic digestion method of Zhang et al. [51] was used to examine digestibility of yoghurt samples without modification. The sample (2 mL) was collected before digestion, after oral (5 min), gastric (120 min) and intestinal (180 min) phases. A total of 305 min and 4 sampling time points, for each set of 7 formulations (28 samples) were achieved. Each sample was transferred into a centrifuge tube and snap-frozen in liquid nitrogen to stop further digestion. When the tube was defrosted at room temperature it was centrifuged at 2000 RPM for 10 min. The supernatant was transferred into another microcentrifuge tube and centrifuged again at 10,000 RPM for 10 min. The supernatant was stored at −20 °C until further analysis. The volume of samples taken after each digestion phase (2 mL) was subtracted for calculation.

### 3.4. Analysis of Soluble Proteins Using Sodium Dodecyl Sulfate Polyacrylamide Gel Electrophoresis

Protein profiles of control and tamarillo yoghurt samples, before and at the end of three phases of digestion were determined by the sodium dodecyl sulfate polyacrylamide gel electrophoresis (SDS-PAGE) method with 4 gels (before, oral, gastric and intestinal), each with 10 lanes (Figure 1). A 10 µL aliquot of pre-stained protein standard (SeeBlue^®^ Thermo Fisher Scientific) with molecular weights in the range 3–198 kDa was applied to lanes 1, 5 and 10 of each gel.

For the yoghurt samples, 20 µL of each sample: PRE15, PRE10, PRE5, POS15, POS10, POS5 and control (n = 7 for each time point) were each mixed with 10 µL of LDS Sample Buffer (4×) and 10 µL of MilliQ water in an Eppendorf tube. After vortexing for 30 s, the mixture was incubated at 70 °C for 10 min. Then, 20 µL of each sample was carefully added into gel wells. Running buffer was prepared by mixing 20 mL of MES Running Buffer with 380 mL of MilliQ water. The electrophoresis was performed by using a constant voltage (Bio-Rad, PowerPac™ Basic, CA, USA) set at 150 V at room temperature for 40 min. At the completion of electrophoresis, the gel was removed from the cassette and placed in a vessel containing Coomassie gel stain which was prepared by mixing 1.0 g Coomassie Brilliant Blue with 450 mL of 95% methanol, 100 mL of glacial acetic acid and 450 mL of MilliQ water. After staining for 30 min, the gel stain solution was discarded, a gel destain solution containing 100 mL of 95% methanol, 100 mL of glacial acetic acid and 800 mL of MilliQ water were added. The gel was soaked overnight with the destaining solution. During this time, the destain solution was changed for three times. The gel was visually assessed when placed on a flat white surface under natural light according to the method agreed by international consensus [52].

### 3.5. Determination and Quantification of Free Amino Acids (FAAs)

Identification and quantification of free amino acids were implemented according to our previous study [3] without further modification. The LC-ESI-MS/MS equipped with Kinetex C18 column (150 × 2.1 mm, 1.7 μm; Phenomenex, CA, USA) and two Agilent MassHunter software (Qualitative Analysis and Quantitative Analysis for QQQ) (Santa Clara, CA, USA) were used to identify, qualify and quantify FAAs. Quantification of each FAA was carried out using standard calibration curves with a coefficient of correlation >0.99.

### 3.6. Determination and Quantification of Phenolics and Anthocyanins

Identification and quantification of phenolics and anthocyanins were implemented according to our previous study [4] without further modification. Analytical standards of phenolics (gallic acid, ellagic acid, ferulic acid, chlorogenic acid, rutin, kaempferol, kaempferol-3-rutinoside and isorhamnetin-3-rutinoside) and three anthocyanins (cyanidin-3-rutinoside, delphinidin-3-rutinoside and pelargonidin-3-rutinoside) were used for calibration. The LC-ESI-MS/MS was also used to identify polyphenols in yoghurts.

### 3.7. Total Phenolic Content (TPC) and Antioxidant Activity of Yoghurt

The total phenolic content (TPC) of extracts and digests at each stage was determined using Folin–Ciocalteu assay as described in our previous study [4]. Two different methods were used to determine the antioxidant activity, namely cupric ion reducing antioxidant capacity (CUPRAC) and ferric-reducing antioxidant power (FRAP) assays [4]. Results of TPC and antioxidant activity were presented as mg gallic acid equivalent per 100 g of yoghurt (mg GAE/100 g yoghurt) and mg Trolox equivalent antioxidant capacity per 100 g of yoghurt (mg TEAC/100 g yoghurt), respectively.

### 3.8. Statistical Analysis

Statistical analysis was carried out using SPSS 25.0 software (IBM Corp., Armonk, NY, USA). All experiments were carried out in triplicate, and data are reported as mean ± standard deviation. The differences among mean values of concentrations of polyphenols or antioxidant activity and that obtained in the different steps of the digestion were determined by one-way analysis of variance (ANOVA) with Fisher’s (LSD) post hoc test. Statistical significance was set at *p* < 0.05.

## 4. Conclusions

Tamarillo powder was successfully employed either before or after fermentation to produce yoghurts fortified with tamarillo and these were highly nutritious containing essential amino acids, GABA and polyphenols. The availability of amino acids at the end of intestinal digestion was increased by adding tamarillo powder. Protein profiling showed that 10–15% addition of tamarillo powder could improve both proteolytic activity and protein content. At the end of digestion, soluble caseins were totally hydrolyzed in all yoghurt samples. For undigested samples, addition of tamarillo powder led to a dose-dependent increase in FAA content, especially the precursor of GABA as well as the TFAAs, TEFAAs and TNEFAAs. PRE samples showed higher amount of TFAAs (6–26%), TEFAAs (3–6 times) and TNEFAAs (4.6–13%) than the POS ones. The highest content of FAAs and no molecular band from SDS-PAGE after intestinal phase indicated that protein hydrolysis was complete, and these would be absorbable in the small intestine.

For either of the fermentation approaches used, the four major tamarillo polyphenols were significantly retained in the final yoghurt products with high TPC and antioxidant activity in undigested samples. With 10% and 15% of tamarillo added, relatively similar antioxidant activity between POS and PRE samples were observed. The antioxidant capacity of fortified yoghurts from both fermentation processes increased under the influence of intestinal digestion, due to chemical changes of the polyphenols and presence of bioactive amino acids or peptides. The percentage ratio of main polyphenols in digested and undigested yoghurt samples was high indicating high bioaccessibility of these bioactive compounds. The results suggested that yoghurt matrix allowed the protection of some polyphenols from hydrolysis, having become bioaccessible and making absorption and utilization possible. Further research of acceptability, shelf life, and then trials for health effects should be implemented. Additionally, the polyphenol-protein interaction needs further investigation to get a better understanding of effect on content of free polyphenols, antioxidant capacity and bioavailability of polyphenols in yoghurt matrix.

## Figures and Tables

**Table 1:** Concentrations (mg/100 g yoghurt) of total free amino acids in control and tamarillo fortified yoghurts in undigested and after intestinal in vitro digestion.

Yoghurt Samples	Undigested			After Intestinal Digestion		
	TEFAAs	TNEFAAs	TFAAs	TEFAAs	TNEFAAs	TFAAs
Control	1.56 ± 0.31 ^a^	5.42 ± 0.81 ^a^	6.98 ± 1.11 ^a^	722 ± 129 ^a^	599 ± 123 ^a^	1321 ± 251 ^a^
POS5	12.3 ± 0.48 ^b^	238 ± 18.2 ^b^	251 ± 18.7 ^b^	778 ± 43.6 ^b^	918 ± 50.7 ^b^	1695 ± 94.4 ^b^
POS10	20.0 ± 0.58 ^c^	404 ± 4.37 ^c^	424 ± 4.95 ^c^	651 ± 91.6 ^c^	1046 ± 128 ^c^	1697 ± 220 ^b^
POS15	35.2 ± 1.76 ^d^	576 ± 44.6 ^d^	611 ± 46.4 ^d^	691 ± 87.7 ^ac^	1437 ± 169 ^d^	2129 ± 256 ^c^
PRE5	77.4 ± 2.43 ^e^	250 ± 8.01 ^e^	327 ± 10.4 ^e^	1009 ± 190 ^d^	1282 ± 226 ^e^	2290 ± 416 ^c^
PRE10	105 ± 5.75 ^f^	433 ± 15.5 ^f^	538 ± 21.2 ^f^	886 ± 146 ^e^	1550± 255 ^d^	2436 ± 401 ^d^
PRE15	112 ± 9.96 ^g^	538 ± 31.9 ^d^	650 ± 42.0 ^d^	905 ± 128 ^de^	1819 ± 233 ^f^	2724 ± 361 ^e^

Data are expressed as Mean ± SD (n = 3). Different alphabet superscripts indicate statistical difference (*p* < 0.05) across each column. TEFAAs: total essential free amino acids, TNEFAAs: total non-essential free amino acids, TFAAs: total free amino acids. POS5, POS10 and POS15: 5%, 10%, 15% of tamarillo powder was added post fermentation, respectively. PRE5, PRE10 and PRE15: 5%, 10%, 15% of tamarillo powder was added to milk and starter culture prior to fermentation, respectively.

**Table 2:** Concentrations (mg/100 g yoghurt) of key free amino acids in control and tamarillo fortified yoghurts before undigested and after intestinal in vitro digestion.

Yoghurt Samples	Undigested			After Intestinal Digestion		
	L-Aspartic Acid	L-Glutamic Acid	GABA	L-Aspartic Acid	L-Glutamic Acid	GABA
Control	0.16 ± 0.01 ^a^	0.81 ± 0.04 ^a^	0.03 ± 0.00 ^a^	34.0 ± 6.57 ^a^	75.3 ± 13.3 ^a^	0.49 ± 0.40 ^a^
POS5	20.1 ± 1.51 ^b^	171 ± 13.5 ^b^	29.2 ± 2.05 ^b^	59.8 ± 1.90 ^b^	310 ± 11.1 ^b^	39.0 ± 3.19 ^b^
POS10	37.3 ± 0.41 ^c^	285 ± 2.65 ^c^	54.8 ± 0.39 ^c^	68.5 ± 7.15 ^c^	452 ± 33.6 ^c^	61.6 ± 3.21 ^c^
POS15	50.0 ± 2.37 ^d^	409 ± 37.6 ^d^	71.5 ± 2.82 ^d^	93.3 ± 9.95 ^d^	806 ± 91.03 ^d^	73.8 ± 8.32 ^d^
PRE5	19.5 ± 0.43 ^b^	160 ± 5.49 ^e^	26.2 ± 0.80 ^b^	78.6 ± 16.0 ^e^	470 ± 69.1 ^c^	34.3 ± 3.36 ^b^
PRE10	37.9 ± 1.49 ^c^	280 ± 7.88 ^c^	48.6 ± 2.52 ^e^	97.8 ± 18.7 ^d^	750 ± 119 ^d^	66.1 ± 10.17 ^c^
PRE15	47.97 ± 1.92 ^e^	358 ± 20.6 ^f^	61.5 ± 2.96 ^f^	117 ± 19.3 ^f^	1027 ± 123 ^e^	90.6 ± 13.9 ^e^

Data are expressed as Mean ± SD (n = 3). Different alphabets superscripts indicate statistical difference (*p* < 0.05) across each column. TEFAAs: total essential free amino acids, TNEFAAs: total non-essential free amino acids, TFAAs: total free amino acids. POS5, POS10 and POS15: 5%, 10%, 15% of tamarillo powder was added post fermentation, respectively. PRE5, PRE10 and PRE15: 5%, 10%, 15% of tamarillo powder was added to milk and starter culture prior to fermentation, respectively.

**Table 3:** Concentrations (mg/100 g yoghurt) of individual polyphenol in tamarillo fortified yoghurts before in vitro digestion.

Polyphenols/Phases	Before Digestion					
	POS5	POS10	POS15	PRE5	PRE10	PRE15
*Phenolics*						
Gallic Acid	0.044 ± 0.000 ^a^	0.045 ± 0.000 ^a^	0.049 ± 0.001 ^b^	0.044 ± 0.000 ^a^	0.048 ± 0.001 ^b^	0.049 ± 0.001 ^b^
Catechin	0.119 ± 0.085 ^a^	0.165 ± 0.070 ^b^	0.219 ± 0.104 ^c^	0.035 ± 0.014 ^d^	0.113 ± 0.097 ^a^	0.208 ± 0.142 ^c^
Caffeic acid	0.021 ± 0.001 ^a^	0.031 ± 0.003 ^b^	0.050 ± 0.005 ^c^	0.024 ± 0.002 ^a^	0.059 ± 0.014 ^d^	0.060 ± 0.006 ^d^
Chlorogenic acid	4.387 ± 0.274 ^a^	7.005 ± 1.290 ^b^	10.31 ± 0.458 ^c^	4.072 ± 0.031 ^a^	6.052 ± 0.379 ^b^	9.502 ± 0.393 ^c^
Epicatechin	0.695 ± 0.198 ^a^	1.339 ± 0.391 ^b^	1.670 ± 0.470 ^c^	0.837 ± 0.083 ^d^	0.886 ± 0.147 ^d^	1.245 ± 0.185 ^b^
p-Cumaric acid	0.017 ± 0.000 ^a^	0.028 ± 0.001 ^b^	0.048 ± 0.002 ^c^	0.026 ± 0.000 ^b^	0.029 ± 0.001 ^b^	0.040 ± 0.003 ^c^
Ferulic acid	0.003 ± 0.000 ^a^	0.005 ± 0.001 ^b^	0.009 ± 0.002 ^c^	0.028 ± 0.001 ^d^	0.033 ± 0.001 ^e^	0.037 ± 0.004 ^e^
Rutin	0.011 ± 0.000 ^a^	0.023 ± 0.005 ^b^	0.035 ± 0.009 ^c^	0.011 ± 0.000 ^a^	0.022 ± 0.002 ^b^	0.033 ± 0.004 ^c^
Ellagic Acid	0.004 ± 0.000 ^a^	0.003 ± 0.001 ^a^	0.009 ± 0.001 ^b^	0.002 ± 0.000 ^c^	0.002 ± 0.000 ^c^	0.005 ± 0.000 ^a^
Kaempferol-3-rutinoside	2.702 ± 0.075 ^a^	4.208 ± 0.102 ^b^	8.218 ± 0.707 ^c^	2.206 ± 0.010 ^a^	4.290 ± 0.054 ^b^	6.022 ± 0.140 ^d^
Isorhamnetin-3-rutinoside	0.003 ± 0.000 ^a^	0.005 ± 0.001 ^b^	0.008 ± 0.001 ^c^	0.003 ± 0.001 ^a^	0.005 ± 0.001 ^b^	0.007 ± 0.001 ^c^
Kaempferol	0.010 ± 0.001 ^a^	0.011 ± 0.001 ^a^	0.016 ± 0.001 ^b^	0.026 ± 0.003 ^c^	0.028 ± 0.002 ^c^	0.028 ± 0.001 ^c^
*Anthocyanins*						
Delphinidin-3-rutinoside	20.59 ± 1.648 ^a^	23.96 ± 5.714 ^b^	38.22 ± 2.137 ^c^	19.62 ± 0.149 ^a^	21.37 ± 1.807 ^ab^	31.66 ± 1.650 ^d^
Cyanidin-3-rutinoside	0.503 ± 0.066 ^a^	0.685 ± 0.122 ^b^	1.267 ± 0.111 ^c^	0.539 ± 0.025 ^a^	0.626 ± 0.080 ^b^	0.947 ± 0.096 ^d^
Pelargonidin-3-rutinoside	7.733 ± 0.494 ^a^	11.12 ± 1.484 ^b^	23.56 ± 1.063 ^c^	5.100 ± 0.218 ^d^	11.72 ± 0.702 ^b^	16.36 ± 0.685 ^e^

Data are expressed as Mean ± SD (n = 3). Different alphabet superscripts indicate statistical difference (*p* < 0.05) across each row. No polyphenols were detected in the control yoghurt. POS5, POS10, and POS15: 5%, 10%, and 15% tamarillo powder was added post-fermentation, respectively. PRE5, PRE10, and PRE15: 5%, 10%, and 15% tamarillo powder was added to milk and starter culture prior to fermentation, respectively.

**Table 4:** Concentrations (mg/100 g yoghurt) of individual polyphenol in tamarillo fortified yoghurts after in vitro digestion.

Polyphenols/Phases	After Digestion					
	POS5	POS10	POS15	PRE5	PRE10	PRE15
*Phenolics*						
Gallic Acid	0.002 ± 0.001 ^a^	0.002 ± 0.001 ^a^	0.003 ± 0.002 ^b^	0.003 ± 0.001 ^b^	0.008 ± 0.004 ^c^	0.009 ± 0.002 ^c^
Catechin	0.014 ± 0.006 ^a^	0.033 ± 0.010 ^b^	0.036 ± 0.006 ^b^	0.005 ± 0.000 ^c^	0.022 ± 0.008 ^ad^	0.026 ± 0.002 ^d^
Caffeic acid	0.004 ± 0.001 ^a^	0.008 ± 0.003 ^b^	0.021 ± 0.012 ^c^	0.019 ± 0.003 ^c^	0.045 ± 0.015 ^d^	0.048 ± 0.013 ^d^
Chlorogenic acid	1.926 ± 0.034 ^a^	3.767 ± 0.198 ^b^	4.553 ± 0.260 ^c^	1.724 ± 0.045 ^d^	4.507 ± 0.264 ^c^	5.825 ± 0.207 ^e^
Epicatechin	0.679 ± 0.015 ^a^	0.803 ± 0.062 ^b^	0.871 ± 0.185 ^c^	0.793 ± 0.018 ^b^	0.834 ± 0.025 ^bc^	0.835 ± 0.088 ^c^
p-Cumaric acid	0.011 ± 0.006 ^a^	0.012 ± 0.006 ^b^	0.012 ± 0.003 ^a^	0.010 ± 0.005 ^a^	0.011 ± 0.005 ^a^	0.012 ± 0.008 ^b^
Ferulic acid	0.005 ± 0.002 ^a^	0.005 ± 0.001 ^a^	0.008 ± 0.001 ^b^	0.009 ± 0.003 ^b^	0.014 ± 0.003 ^c^	0.014 ± 0.004 ^c^
Rutin	0.006 ± 0.002 ^a^	0.015 ± 0.005 ^b^	0.018 ± 0.005 ^c^	0.005 ± 0.001 ^a^	0.019 ± 0.006 ^c^	0.020 ± 0.004 ^c^
Ellagic acid	0.065 ± 0.011 ^a^	0.073 ± 0.011 ^b^	0.076 ± 0.019 ^b^	0.059 ± 0.009 ^a^	0.064 ± 0.006 ^a^	0.092 ± 0.009 ^c^
Kaempferol-3-rutinoside	0.825 ± 0.092 ^a^	1.880 ± 0.227 ^b^	4.137 ± 0.380 ^c^	1.501 ± 0.249 ^b^	3.647 ± 0.525 ^d^	4.155 ± 0.272 ^c^
Isorhamnetin-3-rutinoside	0.005 ± 0.003 ^a^	0.006 ± 0.002 ^ab^	0.007 ± 0.003 ^b^	0.003 ± 0.001 ^a^	0.004 ± 0.001 ^a^	0.004 ± 0.001 ^a^
Kaempferol	0.018 ± 0.001 ^a^	0.018 ± 0.002 ^a^	0.023 ± 0.005 ^b^	0.018 ± 0.001 ^a^	0.022 ± 0.004 ^b^	0.024 ± 0.001 ^b^
*Anthocyanins*						
Delphinidin-3-rutinoside	1.147 ± 0.010 ^a^	2.802 ± 0.064 ^b^	5.527 ± 0.004 ^c^	4.560 ± 0.008 ^d^	8.159 ± 0.090 ^e^	9.033 ± 0.016 ^f^
Cyanidin-3-rutinoside	0.639 ± 0.024 ^a^	0.641 ± 0.051 ^b^	0.672 ± 0.052 ^c^	0.653 ± 0.210 ^b^	0.667 ± 0.181 ^c^	0.687 ± 0.192 ^c^
Pelargonidin-3-rutinoside	1.601 ± 0.019 ^a^	2.072 ± 0.059 ^b^	4.147 ± 0.092 ^c^	1.740 ± 0.067 ^a^	2.557 ± 0.045 ^d^	2.617 ± 0.009 ^d^

Data are expressed as Mean ± SD (n = 3). Different alphabet superscripts indicate statistical difference (*p* < 0.05) across each row. No polyphenols were detected in the control yoghurt. POS5, POS10, and POS15: 5%, 10%, and 15% tamarillo powder was added post-fermentation, respectively. PRE5, PRE10, and PRE15: 5%, 10%, and 15% tamarillo powder was added to milk and starter culture prior to fermentation, respectively.

## Data Availability

All data is contained within the article and Supplementary Material.

## Supplementary Materials

The following are available online at https://www.mdpi.com/article/10.3390/ijms23052526/s1.
