# Supplementary material for: Effect of In Vitro Gastrointestinal Digestion on Amino Acids, Polyphenols and Antioxidant Capacity of Tamarillo Yoghurts
Source: Int J Mol Sci. 2022 Feb 25;23(5):2526. doi: 10.3390/ijms23052526 (PMC8910476; doi:10.3390/ijms23052526)
Supplement: Supplementary file 1 [file ijms-23-02526-s001.zip › ijms-1599039-SI.pdf]

Table S1. Estimated contributions of Laird's Large tamarillo and milk to nutrient and phytochemical content of fortified yoghurt

Nutrients	Tamarillo pulp (g%)	Tamarillo powder (g%)	Milk (g%)	5% yoghurt (g%)	10% yoghurt (g%)	15% yoghurt (g%)
Water	88.1	3	87.6	83.4	79.1	74.9
Protein	1.2	10.08	3.3	3.64	3.98	4.32
Lipid	0.29	2.44	3.1	3.07	3.03	3.00
Fibre	3	25.21	0	1.26	2.52	3.78
Carbohydrate	3.8	31.93	4.5	5.87	7.24	8.61
**Phytochemicals**		(mg%)	(mg%)	(mg%)	(mg%)	(mg%)
γ–Amino butyric acid (GABA)	n.a	433	n.a	21.7	43.3	65.0
<u>Total Phenolics</u>	n.a	122.26	n.a	6.1	12.2	18.3
Rutin	n.a	0.97	n.a	0.05	0.10	0.15
Kaempferol-3-rutinoside	n.a	50.04	n.a	2.5	5.0	7.5
Chlorogenic acid	n.a	66.35	n.a	3.3	6.6	10.0
<u>Total anthocyanins</u>	n.a	481.37	n.a	24.1	48.1	72.2
Delphinidin-3-rutinoside	n.a	254.76	n.a	12.7	25.5	38.2
Pelargonidin-3-rutinoside	n.a	200.66	n.a	10.0	20.1	30.1
<u>Total phenolic content and antioxidant activity</u>						
TPC (mg GAE%)	n.a	707.04	n.a	35.4	70.7	106.1
CUPRAC (mg TEAC%)	n.a	1312.02	n.a	65.6	131.2	196.8
FRAP (mg TEAC%)	n.a	1004.33	n.a	75.3	150.7	225.9

Contributions of tamarillo compositions were calculated from our previous papers [3,4]. Contributions of milk compositions were calculated from reference [50]. Total Phenolics was calculated from all phenolics determined by LC-MS/MS. TPC was identified by the Folin-Ciocalteu assay. TPC: total phenolic content; CUPRAC: cupric ion-reducing antioxidant capacity; FRAP: ferric reducing ability of plasma; GAE: gallic acid equivalent; TEAC: trolox equivalent antioxidant capacity; n.a: not applicable

Table S2A. Concentrations (mg/100g yoghurt) of free amino acids in control and tamarillo fortified yoghurts, undigested.

Free amino acids	Control	POS5	POS10	POS15	PRE5	PRE10	PRE15
L-Histidine	0.21 ± 0.11^{a}	5.9 ± 0.14^{b}	10.75 ± 0.37^{c}	18.1 ± 1.18^{a}	5.36 ± 0.15^{b}	10.75 ± 0.63^{c}	13.9 ± 1.82^{e}
L-Threonine	0.13 ± 0.03^{a}	0.67 ± 0.06^{b}	0.89 ± 0.01^{c}	1.90 ± 0.03^{d}	3.53 ± 0.14^{e}	5.21 ± 0.16^{f}	5.50 ± 0.35^{f}
L-Lysine	0.28 ± 0.01^{a}	2.50 ± 0.10^{b}	4.18 ± 0.02^{c}	7.26 ± 0.24^{d}	6.92 ± 0.23^{d}	10.6 ± 0.71^{e}	10.9 ± 0.90^{e}
L-Valine	0.10 ± 0.02^{a}	0.57 ± 0.04^{b}	0.85 ± 0.04^{c}	1.36 ± 0.04^{d}	10.2 ± 0.34^{e}	12.3 ± 0.55^{f}	11.3 ± 0.82^{ef}
L-Methionine	0.09 ± 0.00^{a}	0.14 ± 0.00^{b}	0.20 ± 0.01^{c}	0.33 ± 0.05^{d}	1.42 ± 0.12^{e}	2.00 ± 0.28^{f}	2.56 ± 0.52^{f}
L-Leucine	0.06 ± 0.01^{a}	0.48 ± 0.03^{b}	0.62 ± 0.03^{c}	1.47 ± 0.05^{d}	7.31 ± 0.21^{e}	8.99 ± 0.69^{f}	9.50 ± 0.41^{g}
L-Isoleucine	0.18 ± 0.02^{a}	0.86 ± 0.04^{b}	1.13 ± 0.05^{c}	2.25 ± 0.05^{d}	24.3 ± 0.74^{e}	30.7 ± 2.37^{f}	33.0 ± 1.25^{f}
L-Phenylalanine	0.36 ± 0.07^{a}	0.73 ± 0.04^{b}	0.83 ± 0.03^{c}	1.42 ± 0.07^{d}	13.3 ± 0.35^{e}	18.0 ± 2.13^{f}	18.4 ± 0.92^{f}
L-Tryptophan	0.15 ± 0.03^{a}	0.39 ± 0.03^{b}	0.51 ± 0.02^{c}	1.15 ± 0.06^{d}	5.00 ± 0.14^{e}	6.85 ± 0.80^{f}	7.15 ± 0.40^{g}
TEFAAs	*1.56 ± 0.31*^{a}	*12.3 ± 0.48*^{b}	*20.0 ± 0.58*^{c}	*35.2 ± 1.76*^{d}	*77.4 ± 2.43*^{e}	*105 ± 5.75*^{f}	*112 ± 9.96*^{g}
Hydroxy-L-Proline	0.02 ± 0.00^{a}	0.14 ± 0.01^{b}	0.22 ± 0.00^{c}	0.21 ± 0.01^{c}	0.15 ± 0.01^{b}	0.25 ± 0.01^{d}	0.33 ± 0.05^{e}
L-Carnosine	0.06 ± 0.03^{a}	0.06 ± 0.02^{a}	0.07 ± 0.02^{a}	0.29 ± 0.14^{b}	0.11 ± 0.02^{c}	0.11 ± 0.01^{c}	0.20 ± 0.11^{b}
L-Arginine	0.41 ± 0.05^{a}	1.74 ± 0.02^{b}	2.71 ± 0.12^{c}	4.09 ± 0.26^{d}	6.09 ± 0.10^{e}	7.23 ± 0.53^{f}	7.48 ± 0.67^{f}
Ethanolamine	0.41 ± 0.05^{a}	1.64 ± 0.10^{b}	1.74 ± 0.06^{b}	3.31 ± 0.08^{c}	2.29 ± 0.08^{d}	2.78 ± 0.09^{e}	2.81 ± 0.20^{e}
L-Serine	0.10 ± 0.07^{a}	1.50 ± 0.10^{b}	2.98 ± 0.07^{c}	4.13 ± 0.11^{d}	3.88 ± 0.11^{e}	7.17 ± 0.27^{f}	7.49 ± 0.44^{f}
Glycine	0.04 ± 0.04^{a}	0.14 ± 0.02^{b}	0.18 ± 0.00^{c}	0.55 ± 0.05^{d}	1.80 ± 0.05^{e}	3.16 ± 0.11^{f}	3.26 ± 0.34^{f}
Sarcosine	0.01 ± 0.00^{a}	0.01 ± 0.00^{a}	0.01 ± 0.00^{ab}	0.01 ± 0.00^{b}	0.01 ± 0.00^{a}	0.01 ± 0.00^{ab}	0.01 ± 0.00^{b}
L-Aspartic acid	0.16 ± 0.01^{a}	20.1 ± 1.51^{b}	37.3 ± 0.41^{c}	50.0 ± 2.37^{d}	19.5 ± 0.43^{b}	37.9 ± 1.49^{c}	47.97 ± 1.92^{e}
Taurine	0.36 ± 0.06^{a}	0.28 ± 0.02^{b}	0.26 ± 0.00^{c}	0.26 ± 0.02^{bc}	0.24 ± 0.01^{e}	0.22 ± 0.01^{ef}	0.21 ± 0.04^{f}
β-Alanine	< 0.005^{a}	0.32 ± 0.03^{b}	0.58 ± 0.00^{c}	0.61 ± 0.03^{c}	0.30 ± 0.02^{b}	0.57 ± 0.03^{c}	0.79 ± 0.04^{d}
L-Glutamic acid	0.81 ± 0.04^{a}	171 ± 13.5^{b}	285 ± 2.65^{c}	409 ± 37.6^{d}	160 ± 5.49^{e}	280 ± 7.88^{c}	358 ± 20.6^{f}

L-Citrulline	0.13 ± 0.05^{a}	0.17 ± 0.01^{b}	0.22 ± 0.01^{c}	0.28 ± 0.03^{d}	0.19 ± 0.01^{b}	0.23 ± 0.01^{c}	0.26 ± 0.01^{d}
L-Alanine	0.48 ± 0.04^{a}	3.22 ± 0.20^{b}	5.23 ± 0.08^{c}	10.7 ± 0.08^{d}	5.83 ± 0.15^{e}	9.48 ± 0.41^{f}	10.7 ± 0.48^{d}
γ-Aminobutyric acid	0.03 ± 0.00^{a}	29.2 ± 2.05^{b}	54.8 ± 0.39^{c}	71.5 ± 2.82^{d}	26.2 ± 0.80^{b}	48.6 ± 2.52^{e}	61.5 ± 2.96^{f}
L-Proline	1.64 ± 0.25^{a}	7.1 ± 0.41^{b}	10.1 ± 0.37^{c}	17.5 ± 0.67^{d}	11.0 ± 0.38^{e}	17.4 ± 0.74^{d}	19.5 ± 2.01^{f}
β-Amino-isobutyric acid	0.01 ± 0.00^{a}	0.03 ± 0.01^{b}	0.04 ± 0.00^{c}	0.04 ± 0.01^{c}	0.03 ± 0.00^{b}	0.04 ± 0.00^{c}	0.05 ± 0.00^{d}
α-Aminobutyric acid	0.01 ± 0.00^{a}	0.02 ± 0.01^{a}	0.06 ± 0.01^{b}	0.07 ± 0.00^{b}	0.04 ± 0.00^{c}	0.05 ± 0.01^{c}	0.07 ± 0.00^{b}
δ-Hydroxylysine	n.d.	n.d.	n.d.	n.d.	n.d.	n.d.	n.d.
L-Ornithine	0.04 ± 0.00^{a}	0.26 ± 0.02^{b}	0.89 ± 0.02^{c}	0.94 ± 0.02^{d}	0.25 ± 0.01^{b}	0.48 ± 0.04^{e}	0.62 ± 0.03^{f}
Cystathionine	0.02 ± 0.00^{a}	0.10 ± 0.02^{b}	0.10 ± 0.06^{b}	0.10 ± 0.02^{b}	0.04 ± 0.03^{c}	0.04 ± 0.00^{c}	0.06 ± 0.00^{d}
L-Cystine	n.d.	n.d.	< 0.005^{a}	< 0.005^{a}	n.d.	< 0.005^{a}	0.01 ± 0.00^{b}
L-Anserine	0.05 ± 0.00^{a}	0.07 ± 0.00^{b}	0.11 ± 0.02^{c}	0.12 ± 0.03^{c}	0.13 ± 0.02^{d}	0.25 ± 0.11^{e}	0.30 ± 0.16^{f}
L-Tyrosine	0.62 ± 0.12^{a}	1.18 ± 0.09^{b}	1.89 ± 0.08^{c}	2.73 ± 0.12^{d}	11.6 ± 0.29^{e}	16.9 ± 0.92^{f}	16.3 ± 2.02^{f}
L-Homocystine	n.d.	n.d.	n.d.	n.d.	n.d.	n.d.	n.d.
TNEFAAs	*5.42 ± 0.81*^{a}	*238 ± 18.2*^{b}	*404 ± 4.37*^{c}	*576 ± 44.6*^{d}	*250 ± 8.01*^{e}	*433 ± 15.5*^{f}	*538 ± 31.9*^{d}
TFAAs	*6.98 ± 1.11*^{a}	*251 ± 18.7*^{b}	*424 ± 4.95*^{c}	*611 ± 46.4*^{d}	*327 ± 10.4*^{e}	*538 ± 21.2*^{f}	*650 ± 42*^{d}

* n.d: not detected. Data are expressed as Mean ± SD (n = 3). Different alphabets superscripts indicate statistical difference ($p < 0.05$) across each row. POS5, POS10, and POS15: 5, 10, and 15% tamarillo powder was added post-fermentation, respectively. PRE5, PRE10, and PRE15: 5, 10, and 15% tamarillo powder was added to milk and starter culture prior to fermentation, respectively. TEFAAs: total essential free amino acids, TNEFAAs: total non-essential free amino acids, TFAAs: total free amino acids.

Table S2B. Concentrations (mg/100g yoghurt) of free amino acids in control and tamarillo fortified yoghurts after each step of *in vitro* digestion.

Free amino acids/phases	After Oral phase						
	Control	POS5	POS10	POS15	PRE5	PRE10	PRE15
L-Histidine	4.41 ± 1.62^{a}	7.72 ± 2.39^{b}	12.7 ± 1.11^{c}	15.7 ± 2.14^{d}	18.0 ± 3.54^{e}	17.3 ± 4.95^{e}	15.6 ± 2.48^{d}
L-Threonine	6.58 ± 0.44^{a}	4.21 ± 1.77^{b}	4.06 ± 0.93^{b}	4.96 ± 1.90^{b}	10.8 ± 5.18^{c}	7.68 ± 2.49^{ac}	7.10 ± 0.96^{a}
L-Lysine	20.3 ± 2.97^{a}	22.0 ± 8.22^{b}	27.6 ± 6.15^{c}	27.8 ± 3.69^{c}	44.6 ± 17.0^{d}	34.6 ± 11.5^{e}	35.7 ± 2.65^{e}
L-Valine	4.52 ± 0.34^{a}	6.47 ± 1.98^{b}	8.05 ± 1.53^{c}	8.05 ± 1.65^{c}	18.8 ± 5.37^{d}	16.3 ± 5.05^{e}	16.0 ± 1.95^{e}
L-Methionine	4.47 ± 1.45^{a}	4.18 ± 2.92^{a}	4.47 ± 1.52^{a}	3.85 ± 0.69^{b}	10.1 ± 5.97^{c}	5.72 ± 2.13^{d}	6.18 ± 1.40^{d}
L-Leucine	4.14 ± 0.31^{a}	5.76 ± 1.62^{b}	7.88 ± 1.55^{c}	8.29 ± 1.78^{d}	13.4 ± 3.92^{e}	13.2 ± 3.82^{e}	13.6 ± 1.83^{e}
L-Isoleucine	19.4 ± 2.12^{a}	18.0 ± 5.96^{b}	20.2 ± 4.78^{ab}	19.5 ± 3.6^{a}	45.6 ± 18.1^{c}	38.4 ± 12.7^{d}	37.5 ± 4.60^{d}
L-Phenylalanine	13.7 ± 0.69^{a}	18.8 ± 6.09^{cb}	20.6 ± 5.89^{b}	17.2 ± 3.42^{cb}	40.4 ± 19.9^{d}	29.1 ± 9.60^{e}	26.4 ± 4.26^{e}
L-Tryptophan	2.99 ± 0.32^{a}	3.79 ± 1.34^{b}	4.13 ± 1.20^{c}	3.56 ± 0.42^{b}	10.44 ± 5.41^{d}	7.92 ± 2.44^{de}	6.70 ± 1.12^{e}
TEFAAs	*80.6 ± 10.3*^{a}	*90.9 ± 32.3*^{b}	*110 ± 24.7*^{c}	*109 ± 19.3*^{c}	*212 ± 84.3*^{d}	*170 ± 54.7*^{e}	*165 ± 21.3*^{e}
Hydroxy-L-Proline	0.07 ± 0.00^{a}	0.36 ± 0.02^{b}	0.54 ± 0.04^{c}	0.60 ± 0.07^{c}	0.25 ± 0.02^{d}	0.41 ± 0.01^{e}	0.56 ± 0.03^{f}
L-Carnosine	n.d	n.d	n.d	n.d	n.d	n.d	n.d
L-Arginine	1.69 ± 1.24^{a}	5.34 ± 1.74^{b}	12.7 ± 3.80^{c}	9.96 ± 3.68^{d}	18.3 ± 7.58^{e}	10.4 ± 4.78^{d}	12.9 ± 1.30^{c}
Ethanolamine	1.95 ± 0.21^{a}	3.02 ± 0.67^{b}	3.71 ± 0.35^{b}	4.77 ± 0.31^{c}	4.67 ± 1.48^{c}	4.42 ± 1.11^{e}	4.70 ± 0.48^{c}
L-Serine	5.63 ± 0.63^{a}	6.19 ± 2.09^{b}	6.49 ± 1.82^{b}	7.59 ± 1.65^{c}	15.3 ± 5.76^{d}	14.8 ± 4.33^{d}	10.5 ± 1.28^{e}
Glycine	1.28 ± 0.14^{a}	1.89 ± 0.36^{b}	1.98 ± 0.46^{b}	2.32 ± 0.28^{c}	5.78 ± 2.82^{d}	7.08 ± 3.69^{e}	4.59 ± 0.62^{d}
Sarcosine	0.01 ± 0.00^{a}	0.01 ± 0.00^{a}	0.01 ± 0.00^{a}	0.01 ± 0.00^{a}	0.01 ± 0.00^{a}	0.01 ± 0.00^{a}	0.01 ± 0.01^{a}
L-Aspartic acid	1.21 ± 0.21^{a}	20.5 ± 3.95^{b}	40.7 ± 3.42^{c}	49.5 ± 3.51^{d}	34.5 ± 13.8^{e}	43.4 ± 13.4^{c}	52.8 ± 5.02^{d}
Taurine	1.76 ± 0.11^{a}	1.39 ± 0.01^{b}	1.11 ± 0.09^{c}	1.20 ± 0.13^{c}	1.57 ± 0.10^{d}	1.48 ± 0.08^{d}	1.31 ± 0.06^{b}
β-Alanine	0.10 ± 0.01^{a}	1.18 ± 0.03^{b}	1.94 ± 0.08^{c}	1.35 ± 0.17^{d}	0.54 ± 0.02^{e}	0.95 ± 0.09^{b}	1.25 ± 0.07^{bd}

L-Glutamic acid	4.77 ± 0.27^{a}	180 ± 39.0^{b}	370 ± 19.8^{c}	557 ± 45.2^{d}	376 ± 152^{c}	462 ± 139^{e}	572 ± 62.1^{d}
L-Citrulline	0.26 ± 0.02^{a}	0.32 ± 0.08^{b}	0.39 ± 0.06^{b}	0.54 ± 0.16^{c}	0.33 ± 0.02^{b}	0.49 ± 0.05^{bc}	0.42 ± 0.16^{bc}
L-Alanine	5.18 ± 0.60^{a}	5.87 ± 1.47^{b}	8.91 ± 1.88^{c}	11.8 ± 1.68^{d}	13.0 ± 6.13^{de}	14.1 ± 3.93^{e}	14.8 ± 1.31^{e}
γ-Aminobutyric acid	0.16 ± 0.02^{a}	31.0 ± 6.41^{b}	59.1 ± 6.92^{c}	62.3 ± 2.20^{d}	38.2 ± 13.7^{b}	49.8 ± 14.2^{c}	60.5 ± 5.75^{d}
L-Proline	3.23 ± 0.14^{a}	9.50 ± 2.39^{b}	16.7 ± 3.10^{c}	20.0 ± 2.67^{d}	16.6 ± 5.30^{c}	19.6 ± 4.16^{d}	22.6 ± 3.14^{d}
β-Amino-isobutyric acid	0.02 ± 0.00^{a}	0.06 ± 0.01^{b}	0.08 ± 0.03^{c}	0.08 ± 0.01^{c}	0.04 ± 0.02^{d}	0.06 ± 0.03^{b}	0.05 ± 0.01^{b}
α-Aminobutyric acid	0.12 ± 0.00^{a}	0.17 ± 0.02^{b}	0.24 ± 0.01^{c}	0.29 ± 0.02^{d}	0.24 ± 0.03^{c}	0.25 ± 0.03^{cd}	0.28 ± 0.01^{d}
δ-Hydroxylysine	0.02 ± 0.00^{a}	0.42 ± 0.08^{b}	1.04 ± 0.16^{c}	1.22 ± 0.11^{d}	0.18 ± 0.01^{e}	0.81 ± 0.08^{f}	0.80 ± 0.02^{f}
L-Ornithine	0.34 ± 0.01^{a}	1.69 ± 0.10^{b}	2.68 ± 0.12^{c}	2.08 ± 0.18^{d}	0.97 ± 0.04^{e}	1.53 ± 0.16^{b}	1.96 ± 0.08^{d}
Cystathionine	0.17 ± 0.01^{a}	0.10 ± 0.01^{b}	0.08 ± 0.02^{c}	0.13 ± 0.03^{b}	0.06 ± 0.01^{d}	0.08 ± 0.01^{c}	0.06 ± 0.02^{d}
L-Cystine	0.19 ± 0.04^{a}	0.14 ± 0.10^{b}	0.14 ± 0.07^{b}	0.28 ± 0.02^{c}	0.22 ± 0.02^{d}	0.22 ± 0.03^{d}	0.22 ± 0.04^{d}
L-Anserine	0.22 ± 0.10^{a}	0.14 ± 0.10^{b}	0.10 ± 0.11^{b}	0.22 ± 0.08^{a}	0.13 ± 0.04^{b}	0.22 ± 0.09^{a}	0.16 ± 0.09^{b}
L-Tyrosine	7.43 ± 0.08^{a}	8.87 ± 2.97^{a}	11.1 ± 3.68^{b}	9.82 ± 2.39^{ab}	22.8 ± 11.7^{c}	19.2 ± 5.72^{cd}	18.1 ± 3.06^{d}
L-Homocystine	0.09 ± 0.06^{a}	0.24 ± 0.06^{b}	0.35 ± 0.11^{c}	0.36 ± 0.10^{c}	0.35 ± 0.05^{c}	0.39 ± 0.10^{d}	0.30 ± 0.06^{bc}
TNEFAAs	*35.9 ± 3.9^{a}*	*278 ± 61.7^{b}*	*543 ± 41.4^{c}*	*741 ± 69.3^{d}*	*549 ± 221^{c}*	*652 ± 195^{e}*	*781 ± 84.7^{d}*
TFAAs	*117 ± 14.2^{a}*	*369 ± 94.0^{b}*	*653 ± 66.1^{c}*	*849 ± 88.6^{d}*	*762 ± 305^{e}*	*822 ± 250^{d}*	*945 ± 106^{f}*

* n.d.: not detected. Data are expressed as Mean ± SD (n = 3). Different alphabet superscripts indicate statistical difference ($p < 0.05$) across each row. POS5, POS10, and POS15: 5, 10, and 15% tamarillo powder was added post-fermentation, respectively. PRE5, PRE10, and PRE15: 5, 10, and 15% tamarillo powder was added to milk and starter culture prior to fermentation, respectively. TEFAAs: total essential free amino acids, TNEFAAs: total non-essential free amino acids, TFAAs: total free amino acids.

Table S2B. Concentrations (mg/100g yoghurt) of free amino acids in control and tamarillo fortified yoghurts after each step of in vitro digestion (Cont.)

Free amino acids/phases	After Gastric phase						
	Control	POS5	POS10	POS15	PRE5	PRE10	PRE15
L-Histidine	4.35 ± 1.53[a]	11.8 ± 5.17[b]	11.3 ± 3.35[b]	17.5 ± 3.22[c]	11.6 ± 3.07[b]	13.7 ± 0.47[d]	16.9 ± 4.82[c]
L-Threonine	13.1 ± 1.01[a]	17.2 ± 2.97[b]	13.4 ± 3.46[a]	14.1 ± 1.96[a]	19.6 ± 1.35[c]	18.4 ± 1.82[bc]	19.7 ± 3.16[c]
L-Lysine	16.1 ± 1.60[a]	21.6 ± 5.29[b]	18.3 ± 3.54[ab]	22.0 ± 3.12[b]	27.5 ± 2.40[c]	29.2 ± 1.20[c]	35.1 ± 4.43[d]
L-Valine	16.5 ± 1.13[a]	22.3 ± 4.26[b]	17.5 ± 5.34[a]	17.7 ± 2.12[a]	28.4 ± 1.98[c]	27.6 ± 1.97[c]	30.5 ± 5.01[d]
L-Methionine	8.56 ± 3.45[a]	11.9 ± 2.53[b]	9.53 ± 5.93[ab]	8.27 ± 0.34[a]	11.0 ± 2.47[b]	12.9 ± 2.81[c]	11.2 ± 5.22[b]
L-Leucine	11.2 ± 0.77[a]	15.0 ± 2.78[b]	12.2 ± 2.55[ab]	12.9 ± 1.29[ab]	18.5 ± 0.70[c]	19.6 ± 1.74[c]	22.1 ± 2.51[d]
L-Isoleucine	58.2 ± 2.92[a]	72.6 ± 11.7[b]	56.3 ± 15.8[a]	54.6 ± 4.69[a]	84.0 ± 6.68[cd]	80.5 ± 5.75[c]	88.7 ± 16.7[d]
L-Phenylalanine	51.4 ± 5.86[a]	66.6 ± 13.6[b]	51.2 ± 11.0[a]	48.7 ± 4.18[c]	69.0 ± 3.45[c]	62.5 ± 5.53[b]	63.5 ± 13.1[b]
L-Tryptophan	15.3 ± 1.79[a]	18.8 ± 3.83[b]	13.4 ± 4.93[c]	12.3 ± 1.09[c]	19.4 ± 0.57[b]	16.8 ± 1.27[a]	16.4 ± 4.07[a]
TEFAAs	*195 ± 20.1[a]*	*258 ± 52.1[b]*	*203 ± 55.9[c]*	*208 ± 22.0[c]*	*289 ± 22.7[d]*	*281 ± 22.6[d]*	*304 ± 59.0[e]*
Hydroxy-L-Proline	0.33 ± 0.03[a]	0.59 ± 0.05[b]	1.03 ± 0.21[c]	0.89 ± 0.11[d]	0.51 ± 0.08[b]	0.85 ± 0.10[d]	0.98 ± 0.24[cd]
L-Carnosine	n.d.	n.d.	n.d.	n.d.	n.d.	n.d.	n.d.
L-Arginine	12.6 ± 7.75[a]	3.91 ± 1.37[b]	2.55 ± 1.63[c]	6.19 ± 1.35[d]	6.07 ± 2.72[d]	8.91 ± 1.24[e]	13.9 ± 7.89[a]
Ethanolamine	2.90 ± 0.06[a]	4.34 ± 0.84[b]	4.29 ± 0.55[b]	6.07 ± 0.57[c]	4.90 ± 0.34[b]	5.63 ± 0.60[bc]	6.55 ± 0.65[d]
L-Serine	14.7 ± 2.69[a]	20.7 ± 4.48[b]	16.8 ± 4.41[c]	16.7 ± 3.25[c]	21.3 ± 1.81[b]	22.7 ± 1.50[d]	23.9 ± 2.58[d]
Glycine	13.3 ± 0.88[a]	15.8 ± 2.36[b]	12.9 ± 3.35[a]	12.6 ± 1.22[a]	17.2 ± 0.28[c]	17.1 ± 1.09[c]	17.2 ± 1.77[c]
Sarcosine	0.01 ± 0.00[a]	0.04 ± 0.01[b]	0.14 ± 0.02[c]	0.10 ± 0.08[c]	0.12 ± 0.00[c]	0.04 ± 0.06[b]	0.01 ± 0.00[a]
L-Aspartic acid	25.7 ± 2.38[a]	58.7 ± 10.1[b]	61.9 ± 14.3[b]	73.7 ± 8.51[c]	56.9 ± 4.00[b]	70.1 ± 4.95[c]	91.4 ± 13.8[d]
Taurine	10.0 ± 0.76[a]	8.26 ± 0.62[b]	9.04 ± 1.10[c]	9.16 ± 0.52[ac]	9.37 ± 0.57[ac]	9.75 ± 1.03[a]	9.49 ± 1.21[ac]

β-Alanine	0.29 ± 0.03^{a}	1.22 ± 0.03^{b}	2.38 ± 0.15^{c}	1.64 ± 0.04^{b}	0.74 ± 0.07^{d}	1.27 ± 0.10^{b}	1.67 ± 0.17^{b}
L-Glutamic acid	45.7 ± 2.11^{a}	300 ± 41.4^{b}	410 ± 128^{c}	650 ± 64.1^{d}	372 ± 33.6^{c}	552 ± 41.0^{e}	771 ± 98.9^{f}
L-Citrulline	0.79 ± 0.11^{a}	0.68 ± 0.09^{b}	0.89 ± 0.16^{c}	0.98 ± 0.03^{d}	0.88 ± 0.05^{c}	1.05 ± 0.08^{d}	0.70 ± 0.55^{b}
L-Alanine	20.1 ± 2.09^{a}	27.8 ± 4.34^{b}	23.4 ± 6.70^{c}	26.7 ± 3.23^{bc}	30.0 ± 1.07^{b}	30.5 ± 1.65^{b}	35.6 ± 4.62^{d}
γ-Aminobutyric acid	0.56 ± 0.13^{a}	42.3 ± 4.78^{b}	60.4 ± 11.5^{c}	62.5 ± 9.30^{c}	31.1 ± 3.01^{d}	52.7 ± 1.81^{e}	74.5 ± 11.3^{f}
L-Proline	6.82 ± 0.18^{a}	13.2 ± 1.99^{b}	14.4 ± 4.77^{b}	19.1 ± 1.45^{c}	14.9 ± 1.35^{b}	20.9 ± 1.61^{c}	26.2 ± 3.84^{d}
β-Amino-isobutyric acid	0.05 ± 0.01^{a}	0.08 ± 0.02^{b}	0.10 ± 0.02^{c}	0.08 ± 0.02^{b}	0.11 ± 0.02^{c}	0.16 ± 0.02^{d}	0.11 ± 0.03^{c}
α-Aminobutyric acid	0.13 ± 0.02^{a}	0.16 ± 0.01^{b}	0.27 ± 0.02^{c}	0.19 ± 0.05^{b}	0.21 ± 0.01^{b}	0.29 ± 0.01^{c}	0.19 ± 0.15^{b}
δ-Hydroxylysine	0.03 ± 0.01^{a}	0.05 ± 0.02^{b}	0.07 ± 0.02^{c}	0.08 ± 0.04^{c}	0.05 ± 0.01^{b}	0.06 ± 0.00^{b}	0.08 ± 0.00^{c}
L-Ornithine	3.51 ± 0.26^{a}	4.51 ± 0.18^{b}	6.25 ± 0.25^{c}	5.64 ± 0.26^{d}	4.60 ± 0.09^{b}	4.88 ± 0.35^{b}	5.44 ± 0.28^{d}
Cystathionine	0.28 ± 0.06^{a}	0.22 ± 0.04^{b}	0.23 ± 0.02^{b}	0.22 ± 0.07^{b}	0.22 ± 0.05^{b}	0.22 ± 0.04^{b}	0.30 ± 0.02^{a}
L-Cystine	7.71 ± 0.60^{a}	6.67 ± 0.54^{b}	7.69 ± 0.57^{a}	7.64 ± 0.13^{a}	7.39 ± 0.14^{c}	7.20 ± 0.35^{c}	7.53 ± 0.42^{a}
L-Anserine	6.82 ± 0.29^{a}	6.17 ± 0.25^{b}	8.04 ± 0.57^{c}	7.29 ± 0.74^{d}	7.29 ± 0.41^{d}	6.59 ± 0.97^{a}	7.00 ± 0.71^{c}
L-Tyrosine	29.8 ± 2.70^{a}	39.1 ± 8.29^{b}	30.4 ± 9.95^{a}	27.5 ± 2.61^{c}	41.3 ± 1.92^{d}	38.6 ± 3.04^{b}	39.5 ± 8.11^{bd}
L-Homocystine	0.28 ± 0.07^{a}	0.23 ± 0.04^{b}	0.23 ± 0.02^{b}	0.30 ± 0.07^{a}	0.29 ± 0.07^{a}	0.36 ± 0.11^{c}	0.26 ± 0.07^{ab}
TNEFAAs	*202 ± 23.2*^{a}	*555 ± 83.6*^{b}	*673 ± 188*^{c}	*935 ± 97.8*^{d}	*627 ± 51.7*^{c}	*852 ± 61.8*^{d}	*1133 ± 157*^{e}
TFAAs	*397 ± 43.3*^{a}	*813 ± 136*^{b}	*876 ± 244*^{b}	*1143 ± 120*^{c}	*916 ± 74.4*^{d}	*1134 ± 84.3*^{c}	*1437 ± 216*^{e}

* n.d: not detected. Data are expressed as Mean ± SD (n = 3). Different alphabets superscripts indicate statistical difference ($p < 0.05$) across each row. POS5, POS10, and POS15: 5, 10, and 15% tamarillo powder was added post-fermentation, respectively. PRE5, PRE10, and PRE15: 5, 10, and 15% tamarillo powder was added to milk and starter culture prior to fermentation, respectively. TEFAAs: total essential free amino acids, TNEFAAs: total non-essential free amino acids, TFAAs: total free amino acids.

Table S2B. Concentrations (mg/100g yoghurt) of free amino acids in control and tamarillo fortified yoghurts after each step of *in vitro* digestion (Cont.)

Free amino acids/phases	After Intestinal phase						
	Control	POS5	POS10	POS15	PRE5	PRE10	PRE15
L-Histidine	29.3 ± 7.75[a]	30.1 ± 6.95[a]	28.8 ± 10.7[a]	37.1 ± 12.41[b]	44.3 ± 12.7[c]	38.5 ± 6.98[b]	42.9 ± 6.72[c]
L-Threonine	32.4 ± 6.11[ab]	34.8 ± 1.49[a]	29.6 ± 5.37[b]	32.0 ± 3.87[ab]	52.6 ± 13.8[c]	43.0 ± 6.95[d]	43.8 ± 6.34[d]
L-Lysine	177 ± 27.7[a]	194 ± 10.1[b]	164 ± 13.2[a]	179 ± 18.8[a]	236 ± 30.3[cd]	229 ± 40.6[c]	241 ± 40.9[d]
L-Valine	51.6 ± 11.0[ab]	55.5 ± 1.44[a]	46.2 ± 8.32[b]	48.2 ± 4.53[b]	80.6 ± 17.8[c]	69.9 ± 12.0[d]	70.9 ± 9.61[d]
L-Methionine	13.7 ± 5.40[a]	13.0 ± 1.18[a]	10.4 ± 1.85[b]	17.4 ± 3.81[c]	31.2 ± 19.1[d]	12.2 ± 2.26[a]	13.7 ± 4.36[a]
L-Leucine	43.0 ± 8.13[a]	45.6 ± 2.62[a]	37.9 ± 6.34[b]	40.8 ± 3.98[ab]	61.8 ± 14.2[c]	54.7 ± 8.86[d]	57.0 ± 7.22[cd]
L-Isoleucine	191 ± 34.7[a]	200 ± 12.1[a]	163 ± 21.2[b]	169 ± 20.2[b]	251 ± 36.0[c]	221 ± 33.4[ac]	227 ± 31.0[ac]
L-Phenylalanine	133 ± 19.1[a]	148 ± 4.58[b]	126 ± 16.6[a]	125 ± 13.6[a]	179 ± 29.3[c]	162 ± 25.4[c]	157 ± 16.3[b]
L-Tryptophan	51.0 ± 8.74[ab]	55.5 ± 3.22[a]	45.2 ± 8.14[b]	43.8 ± 6.51[b]	73.0 ± 17.3[c]	55.7 ± 9.70[a]	53.1 ± 5.44[a]
TEFAAs	*722 ± 129[a]*	*778 ± 43.6[b]*	*651 ± 91.6[c]*	*691 ± 87.7[ac]*	*1009 ± 190[d]*	*886 ± 146[e]*	*905 ± 128[de]*
Hydroxy-L-Proline	0.50 ± 0.04[a]	0.91 ± 0.05[b]	1.60 ± 0.21[c]	1.10 ± 0.08[d]	0.70 ± 0.05[e]	1.15 ± 0.03[d]	1.25 ± 0.17[f]
L-Carnosine	n.d.	n.d.	n.d.	n.d.	n.d.	n.d.	n.d.
L-Arginine	109 ± 44.3[a]	101 ± 27.3[a]	81.7 ± 20.4[b]	91.3 ± 25.3[ab]	168 ± 37.8[c]	155 ± 29.8[c]	111 ± 25.1[a]
Ethanolamine	5.94 ± 0.33[a]	6.93 ± 0.31[b]	7.11 ± 0.48[b]	8.98 ± 0.06[c]	8.21 ± 0.74[c]	9.76 ± 1.21[d]	10.0 ± 0.32[d]
L-Serine	31.5 ± 7.18[a]	32.9 ± 3.75[a]	26.6 ± 4.76[b]	28.6 ± 2.75[b]	63.1 ± 19.9[c]	47.1 ± 8.85[d]	42.8 ± 5.83[d]
Glycine	74.9 ± 10.5[a]	81.0 ± 1.32[b]	69.6 ± 8.21[c]	69.8 ± 7.71[c]	101 ± 17.04[d]	90.4 ± 15.3[e]	86.0 ± 8.64[be]
Sarcosine	0.08 ± 0.06[a]	0.11 ± 0.08[ab]	0.07 ± 0.01[a]	0.13 ± 0.04[b]	0.13 ± 0.01[b]	0.15 ± 0.02[c]	0.10 ± 0.07[ab]
L-Aspartic acid	34.0 ± 6.57[a]	59.8 ± 1.90[b]	68.5 ± 7.15[c]	93.3 ± 9.95[d]	78.6 ± 16.0[e]	97.8 ± 18.7[d]	117 ± 19.3[f]
Taurine	23.1 ± 0.23[a]	23.6 ± 0.64[a]	27.9 ± 3.77[b]	21.8 ± 1.38[c]	22.1 ± 1.49[c]	25.2 ± 0.76[ab]	22.8 ± 1.57[ac]

β-Alanine	0.82 ± 0.02^{a}	1.91 ± 0.09^{b}	2.09 ± 0.17^{c}	3.62 ± 0.35 ^{d}	1.26 ± 0.05^{e}	1.96 ± 0.12^{b}	2.26 ± 0.19^{c}
L-Glutamic acid	75.3 ± 13.3^{a}	310 ± 11.1^{b}	452 ± 33.6^{c}	806 ± 91.03^{d}	470 ± 69.1^{c}	750 ± 119^{d}	1027 ± 123^{e}
L-Citrulline	0.25 ± 0.07^{a}	0.38 ± 0.17^{b}	0.47 ± 0.01^{c}	1.04 ± 0.15^{d}	0.56 ± 0.11^{c}	1.17 ± 0.07^{d}	1.30 ± 0.03^{e}
L-Alanine	43.0 ± 8.16^{a}	47.9 ± 0.85^{b}	42.4 ± 6.69^{a}	47.5 ± 4.42^{b}	70.6 ± 14.9^{c}	60.6 ± 9.94^{c}	66.0 ± 9.14^{c}
γ-Aminobutyric acid	0.49 ± 0.40^{a}	39.0 ± 3.19^{b}	61.6 ± 3.21^{c}	73.8 ± 8.32^{d}	34.3 ± 3.36^{b}	66.1 ± 10.17^{c}	90.6 ± 13.9^{e}
L-Proline	14.4 ± 3.30^{a}	13.8 ± 0.43^{a}	20.2 ± 0.59^{b}	27.9 ± 1.21^{c}	29.4 ± 6.44^{c}	35.1 ± 5.77^{d}	40.3 ± 3.93^{d}
β-Amino-isobutyric acid	0.19 ± 0.02^{a}	0.18 ± 0.03^{a}	0.25 ± 0.06^{b}	0.22 ± 0.03^{ab}	0.20 ± 0.03^{ab}	0.23 ± 0.01^{ab}	0.17 ± 0.07^{b}
α-Aminobutyric acid	1.08 ± 0.21^{a}	1.17 ± 0.21^{a}	1.47 ± 0.21^{b}	1.06 ± 0.24^{a}	1.26 ± 0.04^{ab}	1.26 ± 0.10^{ab}	0.99 ± 0.09^{a}
δ-Hydroxylysine	0.10 ± 0.01^{ab}	0.08 ± 0.01^{a}	0.13 ± 0.04^{b}	0.13 ± 0.02^{b}	0.12 ± 0.01^{b}	0.11 ± 0.05^{ab}	0.10 ± 0.03^{ab}
L-Ornithine	4.32 ± 0.13^{a}	5.53 ± 0.40^{b}	8.56 ± 0.80^{c}	5.81 ± 0.29^{b}	5.43 ± 0.28^{b}	6.32 ± 1.10^{d}	6.54 ± 0.65^{d}
Cystathionine	0.77 ± 0.07^{a}	0.62 ± 0.02^{b}	0.76 ± 0.07^{a}	0.67 ± 0.05^{b}	0.83 ± 0.05^{c}	0.82 ± 0.14^{c}	0.66 ± 0.18^{b}
L-Cystine	16.6 ± 0.77^{a}	16.4 ± 1.68^{a}	20.4 ± 3.92^{b}	15.0 ± 1.83^{c}	16.4 ± 0.92^{a}	17.5 ± 2.92^{ab}	15.9 ± 1.80^{ac}
L-Anserine	17.2 ± 1.99^{ab}	16.0 ± 3.58^{b}	21.4 ± 4.40^{c}	15.5 ± 0.78^{b}	16.3 ± 1.37^{b}	17.8 ± 2.63^{a}	17.4 ± 1.18^{a}
L-Tyrosine	146 ± 24.7^{a}	158 ± 4.09^{b}	132 ± 17.8^{c}	124 ± 12.74^{c}	193 ± 35.5^{d}	166 ± 27.9^{b}	158 ± 17.3^{b}
L-Homocystine	0.06 ± 0.01^{a}	0.14 ± 0.03^{b}	0.31 ± 0.03^{c}	0.23 ± 0.04^{d}	0.11 ± 0.08^{b}	0.19 ± 0.06^{b}	0.20 ± 0.02^{b}
TNEFAAs	*599 ± 123*^{a}	*918 ± 50.7*^{b}	*1046 ± 128*^{c}	*1437 ± 169*^{d}	*1282 ± 226*^{e}	*1550± 255*^{d}	*1819 ± 233*^{f}
TFAAs	*1321 ± 251*^{a}	*1695 ± 94.4*^{b}	*1697 ± 220*^{b}	*2129 ± 256*^{c}	*2290 ± 416*^{c}	*2436 ± 401*^{d}	*2724 ± 361*^{e}

* n.d: not detected. Data are expressed as Mean ± SD (n = 3). Different alphabets superscripts indicate statistical difference ($p < 0.05$) across each row. POS5, POS10, and POS15: 5, 10, and 15% tamarillo powder was added post-fermentation, respectively. PRE5, PRE10, and PRE15: 5, 10, and 15% tamarillo powder was added to milk and starter culture prior to fermentation, respectively. TEFAAs: total essential free amino acids, TNEFAAs: total non-essential free amino acids, TFAAs: total free amino acids.

Table S3A. Concentrations (mg/100g yoghurt) of individual polyphenols in tamarillo fortified yoghurts after oral phase of *in vitro* digestion

Polyphenols	POS5	POS10	POS15	PRE5	PRE10	PRE15
Phenolics						
Gallic Acid	0.009 ± 0.000^{a}	0.009 ± 0.001^{ab}	0.010 ± 0.000^{b}	0.009 ± 0.000^{a}	0.009 ± 0.000^{a}	0.010 ± 0.002^{b}
Catechin	0.011 ± 0.007^{a}	0.163 ± 0.008^{b}	0.187 ± 0.038^{c}	0.005 ± 0.000^{a}	0.013 ± 0.004^{ad}	0.019 ± 0.006^{d}
Caffeic acid	0.006 ± 0.001^{a}	0.014 ± 0.005^{b}	0.014 ± 0.006^{b}	0.005 ± 0.001^{a}	0.009 ± 0.003^{b}	0.028 ± 0.006^{c}
Chlorogenic acid	0.712 ± 0.033^{a}	0.718 ± 0.011^{a}	1.159 ± 0.059^{b}	0.568 ± 0.012^{c}	0.649 ± 0.010^{d}	1.011 ± 0.022^{b}
Epicatechin	0.381 ± 0.033^{a}	0.415 ± 0.072^{b}	0.453 ± 0.036^{b}	0.301 ± 0.042^{c}	0.396 ± 0.016^{a}	0.404 ± 0.046^{b}
p-Cumaric acid	0.008 ± 0.001^{a}	0.008 ± 0.005^{ab}	0.010 ± 0.004^{b}	0.004 ± 0.001^{c}	0.006 ± 0.004^{ac}	0.007 ± 0.001^{ac}
Ferulic acid	0.005 ± 0.002^{a}	0.008 ± 0.005^{ab}	0.009 ± 0.003^{b}	0.008 ± 0.004^{ab}	0.012 ± 0.004^{c}	0.015 ± 0.005^{c}
Rutin	0.015 ± 0.003^{a}	0.016 ± 0.006^{a}	0.018 ± 0.004^{b}	0.010 ± 0.002^{c}	0.012 ± 0.000^{c}	0.021 ± 0.003^{d}
Ellagic Acid	0.016 ± 0.002^{a}	0.021 ± 0.003^{b}	0.024 ± 0.004^{bd}	0.014 ± 0.001^{c}	0.015 ± 0.003^{ac}	0.027 ± 0.007^{d}
Kaempferol-3-rutinoside	2.407 ± 0.402^{a}	3.276 ± 0.234^{b}	4.561 ± 0.434^{c}	2.812 ± 0.344^{a}	3.532 ± 0.184^{b}	4.222 ± 0.146^{c}
Isorhamnetin-3-rutinoside	0.002 ± 0.001^{a}	0.002 ± 0.000^{a}	0.002 ± 0.000^{a}	0.002 ± 0.001^{b}	0.002 ± 0.001^{b}	0.003 ± 0.000^{b}
Kaempferol	0.007 ± 0.001^{a}	0.007 ± 0.002^{a}	0.009 ± 0.003^{b}	0.006 ± 0.001^{a}	0.008 ± 0.001^{ab}	0.010 ± 0.003^{b}
Anthocyanins						
Delphinidin-3-rutinoside	1.785 ± 0.005^{a}	1.934 ± 0.003^{b}	5.899 ± 0.002^{c}	1.892 ± 0.004^{a}	4.042 ± 0.003^{d}	4.371 ± 0.003^{e}
Cyanidin-3-rutinoside	0.143 ± 0.002^{a}	0.146 ± 0.023^{a}	0.147 ± 0.012^{ab}	0.145 ± 0.050^{a}	0.147 ± 0.027^{ab}	0.151 ± 0.033^{b}
Pelargonidin-3-rutinoside	0.573 ± 0.059^{a}	0.962 ± 0.039^{b}	2.132 ± 0.236^{c}	0.627 ± 0.126^{a}	1.026 ± 0.041^{b}	1.902 ± 0.209^{c}

* Data are expressed as Mean ± SD (n = 3). Different alphabets superscripts indicate statistical difference ($p < 0.05$) across each row. No polyphenols were detected in the control yoghurt. POS5, POS10, and POS15: 5, 10, and 15% tamarillo powder was added post-fermentation, respectively. PRE5, PRE10, and PRE15: 5, 10, and 15% tamarillo powder was added to milk and starter culture prior to fermentation, respectively

Table S3B. Concentrations (mg/100g yoghurt) of individual polyphenols in tamarillo fortified yoghurts after gastric phase of *in vitro* digestion

Polyphenols	POS5	POS10	POS15	PRE5	PRE10	PRE15
Phenolics						
Gallic Acid	0.024 ± 0.002^{a}	0.027 ± 0.002^{b}	0.028 ± 0.001^{b}	0.025 ± 0.001^{ab}	0.024 ± 0.000^{a}	0.025 ± 0.001^{ab}
Catechin	0.008 ± 0.001^{a}	0.015 ± 0.005^{b}	0.040 ± 0.010^{c}	0.012 ± 0.003^{b}	0.013 ± 0.004^{b}	0.028 ± 0.010^{d}
Caffeic acid	0.019 ± 0.002^{a}	0.025 ± 0.004^{b}	0.031 ± 0.002^{c}	0.024 ± 0.003^{b}	0.027 ± 0.003^{b}	0.034 ± 0.006^{c}
Chlorogenic acid	3.043 ± 0.130^{a}	3.194 ± 0.152^{b}	5.931 ± 0.291^{c}	2.937 ± 0.133^{a}	3.480 ± 0.142^{d}	5.635 ± 0.218^{c}
Epicatechin	0.427 ± 0.025^{a}	0.500 ± 0.014^{b}	0.500 ± 0.003^{b}	0.428 ± 0.024^{a}	0.508 ± 0.074^{b}	0.535 ± 0.080^{c}
p-Cumaric acid	0.005 ± 0.001^{a}	0.006 ± 0.002^{ab}	0.007 ± 0.002^{b}	0.005 ± 0.001^{a}	0.006 ± 0.002^{ab}	0.006 ± 0.001^{ab}
Ferulic acid	0.003 ± 0.001^{a}	0.004 ± 0.001^{ab}	0.005 ± 0.001^{b}	0.006 ± 0.001^{bc}	0.008 ± 0.004^{c}	0.008 ± 0.003^{c}
Rutin	0.022 ± 0.005^{a}	0.040 ± 0.010^{b}	0.046 ± 0.008^{b}	0.019 ± 0.004^{a}	0.030 ± 0.005^{c}	0.040 ± 0.002^{b}
Ellagic acid	0.026 ± 0.003^{a}	0.029 ± 0.001^{ab}	0.035 ± 0.008^{b}	0.031 ± 0.007^{b}	0.037 ± 0.010^{b}	0.069 ± 0.019^{c}
Kaempferol-3-rutinoside	1.560 ± 0.227^{a}	3.365 ± 0.361^{b}	6.305 ± 0.488^{c}	2.665 ± 0.347^{d}	4.458 ± 0.475^{e}	5.748 ± 0.261^{f}
Isorhamnetin-3-rutinoside	0.002 ± 0.000^{a}	0.005 ± 0.002^{b}	0.007 ± 0.001^{c}	0.003 ± 0.000^{a}	0.003 ± 0.001^{ab}	0.003 ± 0.001^{ab}
Kaempferol	0.009 ± 0.001^{a}	0.010 ± 0.001^{ab}	0.011 ± 0.002^{b}	0.011 ± 0.001^{b}	0.012 ± 0.001^{c}	0.012 ± 0.001^{c}
Anthocyanins						
Delphinidin-3-rutinoside	1.912 ± 0.040^{a}	3.312 ± 0.092^{b}	7.617 ± 0.035^{c}	7.683 ± 0.058^{c}	8.861 ± 0.022^{d}	9.300 ± 0.107^{e}
Cyanidin-3-rutinoside	0.352 ± 0.042^{a}	0.491 ± 0.050^{b}	0.489 ± 0.042^{b}	0.352 ± 0.025^{a}	0.420 ± 0.011^{c}	0.471 ± 0.055^{bc}
Pelargonidin-3-rutinoside	1.722 ± 0.143^{a}	3.200 ± 0.208^{b}	5.835 ± 0.393^{c}	2.388 ± 0.170^{d}	3.720 ± 0.139^{e}	4.678 ± 0.085^{f}

* Data are expressed as Mean ± SD (n = 3). Different alphabets superscripts indicate statistical difference ($p < 0.05$) across each row. No polyphenols were detected in the control yoghurt. POS5, POS10, and POS15: 5, 10, and 15% tamarillo powder was added post-fermentation, respectively. PRE5, PRE10, and PRE15: 5, 10, and 15% tamarillo powder was added to milk and starter culture prior to fermentation, respectively
